# Efficacy of Interventions That Incorporate Mobile Apps in Facilitating Weight Loss and Health Behavior Change in the Asian Population: Systematic Review and Meta-analysis

**DOI:** 10.2196/28185

**Published:** 2021-11-16

**Authors:** Siew Min Ang, Juliana Chen, Jia Huan Liew, Jolyn Johal, Yock Young Dan, Margaret Allman-Farinelli, Su Lin Lim

**Affiliations:** 1 Department of Dietetics National University Hospital Singapore Singapore; 2 School of Life and Environmental Sciences Charles Perkins Centre The University of Sydney Sydney Australia; 3 Science Unit Lingnan University Hong Kong Hong Kong; 4 Joanna Briggs Institute University of Adelaide Adelaide Australia; 5 Department of Medicine National University Hospital Singapore Singapore

**Keywords:** systematic review, meta-analysis, mobile app, obesity, weight loss, Asian, diet, physical activity, adults, mobile phone

## Abstract

**Background:**

Smartphone apps have shown potential in enhancing weight management in Western populations in the short to medium term. With a rapidly growing obesity burden in Asian populations, researchers are turning to apps as a service delivery platform to reach a larger target audience to efficiently address the problem.

**Objective:**

This systematic review and meta-analysis aims to determine the efficacy of interventions that incorporate apps in facilitating weight loss and health behavior change in the Asian population.

**Methods:**

A total of 6 databases were searched in June 2020. The eligible studies included controlled trials in which an app was used in the intervention. The participants were aged 18 years or older and were of Asian ethnicity. A meta-analysis to test intervention efficacy, subgroup analyses, and post hoc analyses was conducted to determine the effects of adding an app to usual care and study duration. The primary outcome was absolute or percentage weight change, whereas the secondary outcomes were changes to lifestyle behaviors.

**Results:**

A total of 21 studies were included in this review, and 17 (81%) were selected for the meta-analysis. The pooled effect size across 82% (14/17) of the randomized controlled trials for weight change was small to moderate (Hedges *g*=–0.26; 95% CI –0.41 to –0.11), indicating slightly greater weight loss achieved in the intervention group; however, this may not be representative of long-term studies (lasting for more than a year). Supplementing multicomponent usual care with an app led to greater weight loss (Hedges *g*=–0.28; 95% CI –0.47 to –0.09). Asian apps were largely culturally adapted and multifunctional, with the most common app features being communication with health professionals and self-monitoring of behaviors and outcomes.

**Conclusions:**

More evidence is required to determine the efficacy of apps in the long term and address the low uptake of apps to maximize the potential of the intervention. Future research should determine the efficacy of each component of the multicomponent intervention to facilitate the designing of studies that are most effective and cost-efficient for weight management.

**Trial Registration:**

PROSPERO CRD42020165240; https://tinyurl.com/2db4tvn6

## Introduction

### Background

Asian countries typically have lower rates of obesity than Western countries [1,2]. However, globalization has contributed to rapid increases in Asian obesity rates over the last 10-15 years such that between 20% and 35% of Asian adults are overweight or obese [2,3]. Higher body fat percentage, prominent central adiposity, and possible genetic factors predispose Asians to insulin resistance, type 2 diabetes, and cardiovascular diseases that further aggravate the health care burden [4,5], with direct health care costs estimated to be US $100 billion in Asian countries alone [3]. As part of the global action plan for the prevention and control of noncommunicable diseases, the World Health Organization has recommended a focus on improving lifestyle behaviors, including adopting a healthy diet and increasing physical activity to modify the risk factors for obesity and noncommunicable diseases [6].

With the exponential growth of mobile technology in the past decade, researchers have explored the potential of digital health interventions using mobile apps as a service delivery platform to reach a larger target audience [7,8]. This is particularly promising in Asian countries where smartphone adoption is estimated to reach 84% by 2025 [9,10]. The ubiquity, accessibility, multifunctionality, and scalability of apps for health intervention provide health care professionals and researchers an unprecedented avenue for treating, monitoring, and interacting with patients en masse remotely [11,12]. With technological advancement, the number of health- and fitness-related apps targeted at behavioral change has burgeoned, with at least 325,000 apps available on the commercial market in 2017 [13].

To date, several systematic reviews on smartphone efficacy to improve weight and health have concluded that interventions that incorporate apps show potential in weight management as well as in improving diet, physical activity, and chronic disease outcomes and are acceptable in the short to medium term [7,8,14-17]. However, most of the studies included in these systematic reviews have been focused on Western populations. Given the differences in genetics, culture, lifestyle, health beliefs, and health-seeking behaviors between Asian and White populations [5,18,19], it is important to assess if interventions that incorporate apps are efficacious in achieving weight loss in Asians before considering them as part of a national strategy to combat obesity.

### Objective

The aim of this review is to systematically synthesize evidence to address this gap in the literature and provide recommendations for future studies. The primary outcome of this review was absolute or percentage weight loss or other surrogate measures of body fat composition such as BMI or waist circumference. The secondary outcomes included dietary intake, physical activity, self-efficacy with regard to implementing healthy lifestyle behaviors, and user engagement with apps.

## Methods

### Literature Search

The review protocol was prospectively registered (PROSPERO ID: CRD42020165240), with modifications made over the course of the review (Multimedia Appendix 1), and conducted according to the PRISMA (Preferred Reporting Items for Systematic Reviews and Meta-Analyses) guidelines (Multimedia Appendix 2) [20]. Systematic searches were conducted in June 2020 across 6 databases: MEDLINE, CINAHL, Embase, PsycINFO, Global Health, and the Cochrane Central Register of Controlled Trials. The search strategy incorporated Medical Subject Headings, keywords, and free-text search terms. The search terms included app*, application*, mobile app*, *smartphone*, *mHealth*, *weight loss*, *weight change*, *body mass index change*, and Asian*. A sample search for MEDLINE is detailed in Multimedia Appendix 3. *BMC Proceedings*, ProQuest Dissertations & Theses, and Google Scholar were searched for conference proceedings, dissertations, and any unpublished gray literature, whereas the ISRCTN registry, ClinicalTrials.gov, and the World Health Organization International Clinical Trials Registry Platform were queried for eligible clinical trials and research. Reference lists of the eligible studies and review articles were also manually searched for additional papers that warranted inclusion. In addition, a filtered search of the *Journal of Medical Internet Research* and *JMIR mHealth and uHealth* was conducted to locate papers that were published before the respective journals were indexed in MEDLINE.

### Inclusion Criteria and Study Selection

Studies were included if they were randomized controlled trials (RCTs), quasi-randomized trials, or nonrandomized controlled trials (non-RCTs). Interventions with no control group, before-and-after interventions, and observational studies (cohort, case-control, cross-sectional, and ecological) were excluded. To be included in this review, studies needed to use a mobile app either in a single-component (ie, standalone use of apps) or multicomponent (ie, apps as part of an intervention with other components, eg, face-to-face consultation, phone calls, or email reviews) intervention. Given that app engagement typically declines rapidly by the second month [21], this was chosen as the minimum study duration to ensure that the intervention effects of app use and longer-term outcomes could be assessed. Participants had to be aged 18 years or older and of Asian ethnicity. Studies were excluded if participants were reported to have eating disorders or mental health conditions, bariatric surgery, or were within the pregnancy or postpartum period. This review was limited to research published in the English language and from 2008 to date because 2008 was the year in which smartphone apps emerged [22].

### Data Extraction

All titles and abstracts of the retrieved records were independently screened by 2 reviewers (SMA and JC) to identify the records that potentially met the inclusion criteria. Relevant full-text articles were retrieved and independently assessed by both reviewers using the complete inclusion and exclusion criteria. Both reviewers independently extracted data from the articles based on a standardized data extraction form, including study characteristics (author, year, country, study design, study aims, sample number, attrition rate, disease group, conflict of interest, and funding), intervention characteristics (intervention type, duration, app type, app features, and cultural adaptations), and predefined outcomes. The level of agreement between the reviewers for the main stages of screening were assessed using the Cohen κ coefficient. Discrepancies were discussed and resolved between the reviewers. Any missing data or further information required was requested by email from the corresponding authors, with a follow-up reminder sent after 2 months.

### Study Quality Assessment

The Cochrane Collaboration Risk-of-Bias Tool [23] and the Risk of Bias in Non-Randomized Studies of Interventions Tool [24] were implemented independently by 2 reviewers (SMA and JC) to assess the risk of bias in the RCTs and non-RCTs, respectively. Discrepancies were discussed and resolved through a third reviewer (JJ). Each domain of the RCTs received an evaluation of low, moderate, or high risk, whereas the non-RCTs were judged as having low, moderate, serious, or critical risk.

### Outcomes

The primary outcome of this review was absolute (kg) or percentage weight change. Other surrogate measures of body weight, such as BMI (kg/m^2^) and waist circumference (cm), were also included. The secondary outcomes included app use and changes to lifestyle behaviors, including diet, physical activity, and self-efficacy for implementing healthy behaviors.

### Data Analysis

The effect sizes used in the meta-analysis were Hedges *g* values calculated from the mean differences in outcomes (ie, changes in absolute or percentage weight, BMI, or waist circumference before and after treatment) between the treatment arms. Separate analyses were conducted for the RCTs and non-RCTs; studies without a standardized mean and SD were excluded from the meta-analysis. A unique study identifier was assigned to each intervention-control pair included in the meta-analysis (Multimedia Appendix 4 [25-41]).

Random effects models, which control for heterogeneity between studies, were used to fit the Hedges *g* scores. To account for within-study dependencies, comparisons were made separately for each outcome, namely, changes in absolute or percentage weight, BMI, and waist circumference. For studies with multiple time points reported, only the final outcome within the active intervention was included to avoid pseudoreplication. Furthermore, the overall data were also divided into 2 subsets comprising single-component (ie, standalone use of apps) and multicomponent (ie, apps as a part of an intervention with other components) studies, respectively, and analyzed separately. Subsequently, subgroup analyses were conducted to analyze the effects of adding an app to usual care (intervention group) compared with usual care alone (control group).

The possible moderating effect of study duration was also tested using moderation analysis, after which the possible differences in intervention outcomes in studies conducted over 3 months or less versus studies lasting for longer than 3 months were assessed post hoc. These were done as a preliminary assessment of the importance of app engagement levels on study outcomes because adherence to app use generally tails off after 2 to 3 months [8,11,42,43]. The post hoc analyses were conducted in lieu of a more robust meta-regression approach given the dearth of quantifiable app engagement data.

Heterogeneity among the studies for each comparison was assessed using the I^2^ statistic, with values of 30% to 60%, 50% to 90%, and 75% to 100% considered to indicate moderate, substantial, and considerable levels of heterogeneity, respectively [44]. Publication bias was also assessed using a funnel plot. All analyses were conducted using the *metafor* package (2.4.0) in the R statistical environment (R Foundation for Statistical Computing) [45,46].

## Results

### Study and Sample Characteristics

**Figure 1:**
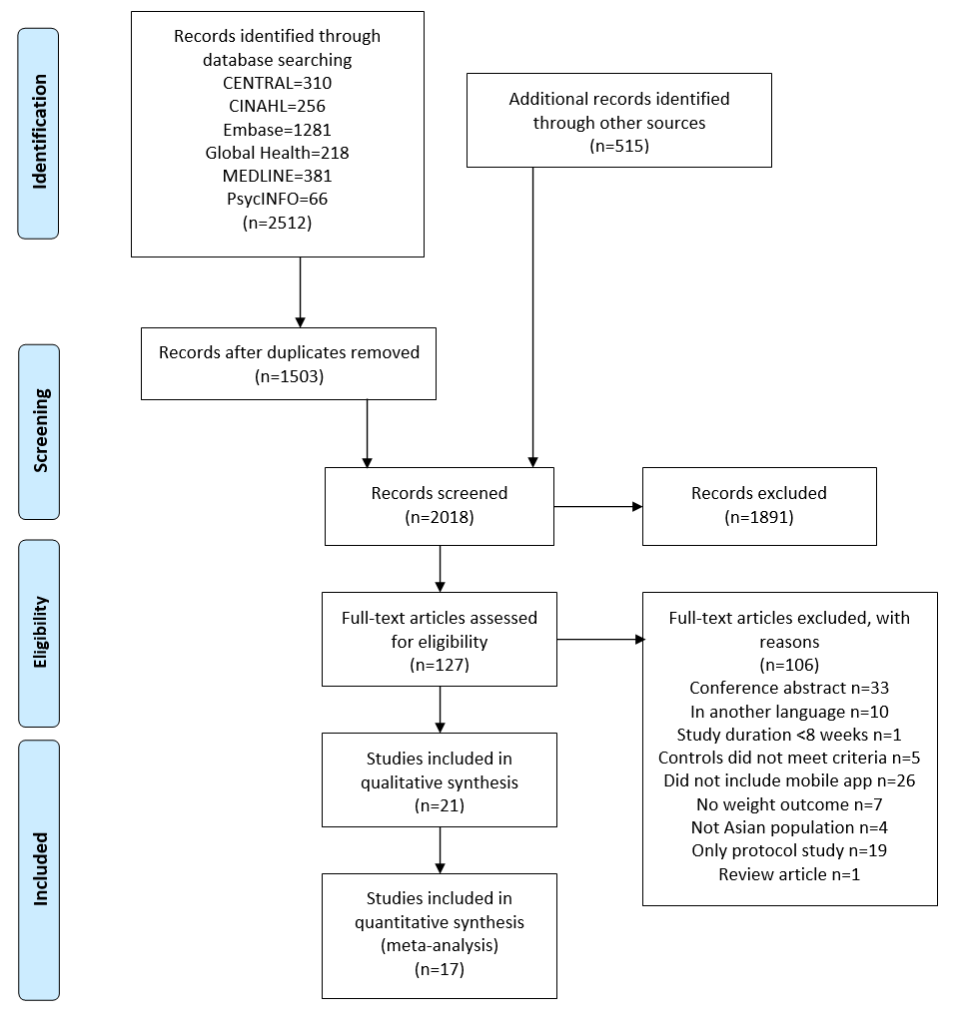
PRISMA (Preferred Reporting Items for Systematic Reviews and Meta-analyses) flow diagram. CENTRAL: Cochrane Central Register of Controlled Trials. CINAHL: Cumulative Index to Nursing and Allied Health Literature.

A total of 3027 electronic records were identified through the search strategy and, after removal of duplicates, 2018 (66.67%) titles were screened. From these 2018 records, 127 (6.29%) full-text articles were retrieved (Figure 1). Of the 127 papers, 21 (16.5%) met all inclusion criteria for the systematic literature review [25-41,47-50]. Of these 21 papers, 17 (81%) were included in the meta-analysis [25-41]. The Cohen κ coefficients for the initial screening stage of titles and abstracts and the full-text screening stage were 0.72 (substantial agreement) and 0.92 (almost perfect agreement), respectively, with 97% level of agreement for each screening stage.

Intervention characteristics and descriptions of the studies included in this review are summarized in Table 1 and Table 2, respectively. Of the 21 papers, 17 (81%) were RCTs and 4 (19%) were non-RCTs, with study locations in China [25,37-39,48], Hong Kong [34], India [26,31], Japan [35], Singapore [30], South Korea [27-29,32,33,36,40,50], Taiwan [41,49], and the United States [47]. The total number of participants across the 21 studies was 21,173 (RCTs: 4090 and non-RCTs: 17,083), with a mean age of 45.9 (SD 9.84, range 25.8-60.5) years; on average, 45.1% (SD 20.1) of the participants were women. The mean BMI of the population was 27.1 (SD 2.47, range 23.0-30.5) kg/m^2^. Of the 21 studies, 9 (43%) were conducted among patients who were overweight or obese [28,30-35,47,49], 6 (29%) involved mainly patients with diabetes mellitus—4 (67%) [27,36,40,48] on type 2, whereas 2 (33%) [37,38] included a mix of type 1 and 2—and 3 (14%) included participants from the general population [26,39,41], whereas the remaining 3 (14%) individually targeted patients with metabolic abnormalities [50], coronary heart disease [25], or colorectal polyps [29].

**Table 1:** Characteristics of the interventions incorporating apps included in the review (N=21).

Author (year), country, ethnicity	Study characteristics, sample size (included in analysis)	Participant characteristics	App characteristic (name), cultural adaptation within app	Measured outcomes	Attrition rate (%)
Bender et al [47] (2018), United States, Filipino American	Pilot RCT^a^, Filipino Americans who were overweight or obese and aged ≥18 years at risk of T2DM^b^ or prediabetes, 3 months, n=67	Mean age, years (SD): 41.7 (12.0), mean BMI, kg/m^2^ (SD): 30.5 (4.4), women (%): 52.2	Multicomponent, commercial (Fitbit), English, no	Weight, BMI, waist circumference, FPG^c^, and HbA_1c_^d^ level	5; I^e^: 6; C^f^: 3
Dong et al [48] (2018), China, Chinese	RCT, patients with T2DM aged 18-60 years, 12 months, n=120 (119)	Mean age, years (SD): 42.7 (6.7), BMI ≥25 kg/m^2^: I (%): 48.3, C (%): 42.4, women (%): 47.9	Multicomponent, commercial (WeChat), Chinese, yes	FPG, 2-hour PG^g^, HbA_1c_ level, total self-efficacy score, diet score, exercise score, medication-taking score, blood glucose–monitoring score, foot-care score, and smoking score	0
Dorje et al [25] (2019), China, Chinese	RCT, patients with coronary heart disease aged ≥18 years, 2 months intervention+4 months step-down phase, n=312	Mean age, years (SD): 60.5 (9.2), mean BMI, kg/m^2^ (SD): I: 25.5 (3.0), C: 25.4 (3.5), women (%): 19	Multicomponent, commercial (WeChat), Chinese, yes	Weight, BMI, waist-to-hip ratio, 6-minute walk test, knowledge and awareness of coronary heart disease, PG, lipids, psychosocial well-being, quality of life, smoking status, dietary habits, and physical activity	15; I: 14; C: 16
Kaur et al [26] (2020), India, Indian	Cluster RCT, adults aged 35-70 years, 6 months, n=732	Mean age, years (SD not reported): 52.7, mean BMI, kg/m^2^ (SD): I: 27.03 (4.2), C: 27.45 (4.8), women (%): 76.1	Multicomponent, commercial (WhatsApp), English, Hindi, and Punjabi, emails in English, yes	Weight, BMI, dietary intake changes, ASE^h^ score, BP^i^, FPG, and lipids	9; I: 8; C: 10
Kim et al [27] (2019), South Korea, Korean	Multicenter RCT, stable patients with T2DM aged 19-80 years with HbA_1c_ level between 7% and 10%, 24 weeks, n=191 (172)	Mean age, years (SD): I: 60.0 (8.4), C: 56.7 (9.1), mean BMI, kg/m^2^ (SD): I: 25.5 (3.2), C: 25.8 (4.1), women (%): I: 44, C: 52	Multicomponent, researcher design (mDiabetes), Korean, yes	Weight, body composition, score of the summary of diabetes self-care activities, World Health Organization quality-of-life scale, HbA_1c_ level, lipids, and BP	21
Lee et al [28] (2018), South Korea, Korean	Multicenter pilot RCT, university medical school students who were overweight or obese with metabolic syndrome, 24 weeks, n=422 (324)	Age: ≥20 years, mean BMI, kg/m^2^ (SD): inactive I: 28.8 (2.72), C: 29.1 (3.10), moderately active I: 29.8 (4.39), C: 29.0 (2.46), health-enhancing physically active I: 29.3 (2.90), C: 29.8 (7.12), women (%): I: 52, C: 48	Multicomponent, researcher design (SmartCare), Korean, app is in English, yes	Weight, BMI, waist circumference, body composition, BP, FPG, HbA_1c_ level, and lipids	23; I: 17; C: 30
Lee et al [29] (2019), South Korea, Korean	Pilot RCT, patients aged 20-65 years with colorectal polyps diagnosis within the last 2 years of the study, 3 months, n=65	Mean age, years (SD): I: 49.1 (8.3), C: 50.7 (8.1), mean BMI, kg/m^2^ (SD): I: 26.9 (3.4), C: 24.5 (3.9), women (%): I: 34.4, C: 45.5	Multicomponent, commercial (Noom), English or Korean (NFS^j^), no	Weight, changes in dietary intake through food frequency questionnaire, and Godin leisure-time exercise questionnaire	3; I: 0; C: 6
Lim et al [30] (2020), Singapore, multiracial	RCT, patients who were overweight or obese with nonalcoholic fatty liver disease and were aged 21-70 years, 6 months, n=108	Mean age, years (SD): I: 46.8 (11.1), C: 46.2 (10.1), mean BMI, kg/m^2^ (SD): I: 30.1 (4.0), C: 30.8 (4.8), women (%): I: 42, C: 32	Multicomponent, researcher design (nBuddy), English, yes	Weight, BMI, waist circumference, BP, and liver enzymes	6; I: 9; C: 4
Muralidharan et al [31] (2019), India, Indian	Multicenter RCT, adults who were overweight or obese aged 20-65 years with prediabetes, 12 weeks, n=741 (561)	Mean age, years (SD): I: 37.8 (9.2), C: 37.8 (9.6), mean BMI not reported, only baseline weight was reported, women (%): I: 43.9, C: 42.1	Multicomponent, researcher design (mDiab), English, yes	Weight, target 5% weight loss	24; I: 28; C: 21
Oh et al [32] (2015), South Korea, Korean	Multicenter RCT, adults who were obese aged 20-70 years with BMI≥25 kg/m^2^ and metabolic syndrome diagnosis, 24 weeks, n=422 (334)	Mean age, years (SD): I: 46.78 (13.11), C: 50.35 (14.24), mean BMI, kg/m^2^ (SD): I: 29.42 (3.53), C: 29.40 (3.39), women (%): I: 46.7, C: 51.4	Multicomponent, researcher design (SmartCare), Korean, app is in English, yes	Weight, BMI, waist circumference, body composition, change in diet habit, change in physical activity (IPAQ^k^), and patient satisfaction	21; I: 15; C: 27
Shin et al [33] (2017), South Korea, Korean	Pilot RCT, men who were overweight or obese aged 19-45 years with BMI≥27 kg/m^2^, 12 weeks, n=105 (98)	Mean age, years (SD): 27.8 (5.0), mean BMI, kg/m^2^ (SD): 29.8 (2.7), women: 0	Multicomponent, researcher design (Fit.Life), Korean, app is in English, yes	Weight, BMI, body composition, physical activity changes (IPAQ), calorie intake changes, BP, FPG, lipids, and liver enzymes	7; C: 9; I1: 3; I2: 9
Suen et al [34] (2019), Hong Kong, Chinese	Feasibility RCT, healthy adults who were overweight or obese aged ≥18 years with BMI≥25 kg/m^2^ without ear injuries, 8 weeks, n=59	Mean age, years (SD): 49.15 (10.54), mean BMI, kg/m^2^ (SD): 30.35 (4.53), women (%): 85	Multicomponent, researcher Design (Auricular Acupressure for Weight Reduction version 1), Chinese, yes	Weight, BMI, body composition, waist and hip circumference, blood leptin and adiponectin, fullness rating, and patient satisfaction	10; C: 5; I: 11
Tanaka et al [35] (2018), Japan, Japanese	RCT, adults who were overweight, obese, or abdominally obese aged 20-64 years with cardiometabolic risk factors or metabolic syndrome, 8 weeks intervention+4 weeks postintervention follow-up, n=112	Mean age, years (SD): I: 45.6 (10.2), C: 47.8 (9.3), mean BMI, kg/m^2^ (SD): I: 28.0 (3.3), C: 28.2 (3.0), women (%): 0.9	Single-component, commercial (FiNC), Japanese yes	Weight, waist circumference, BP, lipids, HbA_1c_ level, and obesogenic eating behaviors	28; I: 32; C:18
Yang et al [49] (2017), Taiwan, Chinese	Crossover RCT, patients who were overweight or obese with BMI≥24 kg/m^2^ and metabolic abnormalities, 3 months intervention+crossover 3 months usual care, n=53 (46)	Mean age, years (SD): 33.2 (9.6), mean BMI, kg/m^2^ (SD): I: 27.2 (3.4), C: 30.3 (4.9), women (%): I: 61.5, C: 59.2	Multicomponent, researcher design (self-monitoring app), NFS (Line, social communication app), NFS on app, Chinese and English noted on website, yes	Weight, BMI, waist circumference, change in physical activity, FPG, BP, and lipids	13; I: 19; C: 7
Yang et al [36] (2020), South Korea, Korean	Cluster RCT, adults aged ≥18 years with T2DM for ≥1 year and HbA_1c_ level 7%-10%, 12 weeks, n=247 (239)	Mean age, years (SD): I: 54.1 (10.1), C: 60.6 (10.2), mean BMI, kg/m^2^ (SD): I: 26.3 (3.7), C: 25.7 (3.9), women (%): I: 46.6, C: 53.6	Multicomponent, researcher design (HiCare smart K), Korean, yes	Weight (not mandatory), BMI, waist circumference, BP, lipids, HbA_1c_ level, FPG, Diabetes Treatment Satisfaction Questionnaire, and medication adherence scale	3; I: 3; C: 3
Zhang et al [37] (2019), China, Chinese	RCT, adults aged 18-65 years, diagnosed with diabetes for more than 6 months and with HbA_1c_ level ≥8%, 6 months, n=234 (194)	Mean age, years (SD): 53 (11), mean BMI, kg/m^2^ (SD): 25.03 (3.36), women (%): 38	Multicomponent, commercial (Welltang), Chinese, yes	Weight, BMI, BP, waist circumference, FPG, HbA_1c_ level, lipids, and liver enzymes	17; C: 19; I1: 14; I2: 18
Zhou et al [38] (2016), China, Chinese	Pilot RCT, adults aged 18-74 years with diagnosed diabetes without severe complications, 3 months, n=100	Mean age, years (SD): I: 55.0 (13.1), C: 53.5 (12.4), mean BMI, kg/m^2^ (SD): I: 23.04 (4.09), C: 23.01 (4.04), women (%): I: 46, C: 40	Multicomponent, commercial (Welltang), Chinese, yes	Weight, BMI, waist and hip circumference, diabetes knowledge and self-care behavior score, HbA_1c_ level, FPG, 2-hour PG, BP, and low-density lipoprotein-c	Not reported
He et al [39] (2017), China, Chinese	Cohort-based non-RCT, general population aged ≥18 years who were keen on weight loss, 6 months, n=15,818 (15,310)	Mean age, years (SD): I: 35.1 (8.5), C: 39.0 (9.5), mean BMI not reported, women (%): I: 66.5, C: 40.5	Single-component, commercial (WeChat), Chinese, yes	Weight and waist circumference	3; I: 4; C: 2
Kim et al [40] (2014), South Korea, Korean	Matched controlled non-RCT, adults aged 20-70 years with T2DM for more than 1 year with HbA_1c_ level 7%-10% at baseline, 3 months, n=73 (70)	Mean age, years (SD): I: 51.8 (10.3), C: 53.8 (9.0), mean BMI, kg/m^2^ (SD): I: 25.0 (3.3), C: 24.9 (3.4), women (%): 49.2	Multicomponent, NFS, likely researcher design (Mobile SmartCare, version 1.0.7), Korean, yes	BMI, BP, HbA_1c_ level, lipids, and patient satisfaction	4; I: 8; C: 0
Kim et al [50] (2019), South Korea, Korean	Cohort-based non-RCT, control: individuals with metabolic abnormalities according to the Adult Treatment Panel III criteria, intervention: individuals recruited from among those who had previously completed a 24-week mobile service program as part of the First Year Public Health Center Mobile Healthcare pilot project, 24 weeks, n=1117	Mean age, years (SD): I: 44.68 (8.22), C: 44.69 (8.22), mean BMI, kg/m^2^ (SD): I: 25.71 (3.34), C: 25.18 (3.48), women (%): I: 50.8, C: 65.5	Multicomponent, researcher design (Public Health Center mHealth app), Korean, yes	Health behavior scores, mini–dietary assessment scores, BP, FPG, triglycerides, high density lipoprotein-c, and waist circumference	Not reported
Wijaya and Widiantoro [41] (2018), Taiwan, Indonesian	Pretest-posttest design, Indonesian international students aged ≥20 years who owned a smartphone with internet access, not participating in other training program, and literate in English, 10 weeks, n=75 (70)	Mean age, years (SD): 25.86 (4.33), mean BMI, kg/m^2^ (SD): 23.25 (3.05), women (%): 45.7	Multicomponent, researcher Design (iNCKU smartphone app) English, yes	Body weight, BMI, BP, physical fitness, physical activity measures (step count, distance covered, caloric expenditure, and time spent on activity), self-efficacy, social support, and outcome expectation	7; I: 8; C: 5

^a^RCT: randomized controlled trial.

^b^T2DM: type 2 diabetes mellitus.

^c^FPG: fasting plasma glucose.

^d^HbA_1c_: glycated hemoglobin.

^e^I: intervention.

^f^C: control.

^g^PG: plasma glucose.

^h^ASE: attitude, social influence, and self-efficacy.

^i^BP: blood pressure.

^j^NFS: not further specified.

^k^IPAQ: International Physical Activity Questionnaire.

**Table 2:** Description and primary outcome of the interventions included in the review (N=21).

Author (year), country, ethnicity, study design	Intervention	Health staff involvement	Control treatment	Change in weight or weight-related outcomes
Bender et al [47] (2018), United States, Filipino American, pilot RCT^a^	Fit&Trim, a DPP^b^-based, culturally adapted, mobile phone–based weight loss lifestyle intervention delivered by Filipino research staff, augmented with tracker Fitbit Zip and private Facebook virtual support group. Provided individual tailored goals for weight, diet, physical activity or steps, and encouraged to monitor lifestyle habits and progress on app	Baseline+5 in-person Fit&Trim education intervention office visits	Active waitlist. Received Fitbit Zip physical activity tracker with 2 education sessions on hepatitis A and B	Weight change (kg) calculated: I^c^: –3.39, C^d^: –0.82, SD or further values not reported, achieved 5% weight loss: I: 36%, C: 6%, large effect of 0.93 (Cohen *d*) reported
Dong et al [48] (2018), China, Chinese, RCT	Received conventional health education with nursing care for diabetes. Patients received multimedia-type diabetes-related knowledge from nurses on app. Able to communicate with educators and friends on app. Phone reviews on app use and hospital physical examination offered	Baseline visit and 6-month and 12-month visits, nurses communicating with patients through WeChat	Conventional health education with nursing care for diabetes	There was no significant difference in BMI between groups at baseline, 6 months, and 12 months. No further values reported
Dorje et al [25] (2019), China, Chinese, RCT	Received usual care+SMART-CR/SP^e^ program. During the intensive phase, participants received 4 educational cartoon modules per week through WeChat. In the step-down phase, participants received only 2 cartoon pictures with key motivational message per week; Cartoon education touched on cardiovascular health and disease, physical activity, healthy nutritional advice, support for medication adherence, psychological well-being, and modification risk factors; Individualized feedback, recommendations, and remote supervision were provided based on regular reviews of monitoring data. Coach support available on app for health and lifestyle advice. Additional alerts and WeChat messages were sent when measurements were outside target blood pressure or steps	Baseline, 2-month, and 6-month visits for measurements and assessments by blinded researchers. Remote supervision through messages, telemonitoring, feedback, and video calls from coaches as necessary based on regular reviews of data	Standard care provided by doctors with a brief education carried out by a nurse. Medication management and ad hoc review visits to a cardiologist or other health care providers according to the patient’s self-assessment of their own cardiovascular health; WeChat used for sending review visit reminders, did not receive any form of health information or intervention	BMI change (kg/m^2^), mean (SD), baseline: I: 25.5 (3.0), C: 25.4 (3.5), 2 months: I: 25.0 (2.9), C: 25.2 (3.2), between-groups *P*=.64, 6 months: I: 24.9 (3.5), C: 24.5 (3.2), between-groups *P*=.14; Waist circumference change (cm), mean (SD), baseline: I: 0.9 (0.1), C: 0.9 (0.1), 2 months: I: 0.9 (0.1), C: 0.9 (0.0), between-groups *P*=.36, 6 months: I: 0.9 (0.0), C: 0.9 (0.1), between-groups *P*=0.95
Kaur et al [26] (2020), India, Indian, cluster RCT	Provision of *SMART Eating* kit, which included a kitchen calendar, dining table mat, and measuring spoons. Received weekly review information through SMS, email, social networking app (WhatsApp), and *SMART Eating* website; Fortnightly addition of diet and health-related content to website, quizzes, and a web-based help assistant to ask questions	Single home visit to provide education and guide family champions on how to use the different components of the intervention; No further face-to-face interaction. Further advice and supervision was conducted through the website	Pictorial pamphlet on the dietary recommendations with information written in Hindi language. Asked to read the pamphlet in their own time, make changes to their diet accordingly, and convey the same information to their family members; Same educational content and materials were offered to intervention group	Weight change (kg), mean (95% CI; SD not reported), 6 months: I: –0.42 (–0.8 to –0.1) *P*=.01, C: 0.24 (–0.1 to 0.6) *P*=.14, net change between groups: –0.66 (–1.1 to –0.2), *P*=.01
Kim et al [27] (2019), South Korea, Korean, multicenter RCT	Participants were divided into 4 groups based on antidiabetic treatment. Nil baseline education. Provided individualized targets for diet and physical activity at baseline. Encouraged to monitor blood glucose levels and lifestyle habits on app and informed that physicians can view and monitor their progress through telemonitoring. App provided immediate feedback according to an algorithm when a reading on blood glucose level, food intake, or activity was entered. Detailed information on diet and physical activity was made available through a range of educational and interactive component on app. Social networking service and bulletin board enabled users to share experiences and tips with each other, whereas research staff could answer questions from patients	Baseline, 2 in-person visits (week 12 and week 24), and 2 phone call reviews. Remote supervision and advice provided on app as well	Nil baseline education. Provided logbook to record blood glucose readings, Bluetooth glucometer, test strips, and a printed education booklet. Received 2 in-person visits at 12 weeks and 24 weeks	Weight change (kg), mean (SD), baseline to 6 months: I: 67.7 (11.8) to 67.1 (11.6), *P*=.005, C: 68.4 (13.0) to 68.0 (12.7), *P*=.04
Lee et al [28] (2018), South Korea, Korean, multicenter pilot RCT	Provided with a smartphone equipped with SmartCare app and Bluetooth-enabled bioimpedance analyzer. Instructed to monitor body composition, data were transmitted to the SmartCare system through the app; Health reports were automatically created based on the personal health information of participants according to the clinical decision support system algorithm function of the SmartCare system. Health managers provided prevention, consultation, and educational services remotely to participants based on health reports through messages and weekly emails. Monthly progress evaluation was offered along with in-person consultation with a physician at least once every 2 months (follow-up study by Oh et al [32], 2015)	Baseline, 2-monthly in-person visits with physician. Measurements at baseline, week 12, and week 24. Weekly remote supervision over app	Provided with weighing scale and pedometer and asked to record weight and physical activity steps progress in a diary. Offered 3 in-person visits at baseline, week 12, and week 24. No further visit details	Weight change (kg), mean (SD) 6 months, insufficiently active: C: –0.1 (1.94), *P*=.64, I: –1.6 (3.03), *P*<.001, between-groups *P*=.001, minimally active: C: –0.3 (2.24), *P*=.49, I: –2.5 (3.81), *P*=.001, between-groups *P*=.01, health-enhancing physical activity: C: –1.5 (3.12), *P*<.001, I: –2.6 (3.91), *P*<.001, between-groups *P*=.05
Lee et al [29] (2019), South Korea, Korean, pilot RCT	Received app and taught to use under supervision during first visit without further education. Health-related information, lifestyle recommendations, and feedback sent through app; Encouraged to track lifestyle habits on app, and users may mutually compete and share progress on the bulletin board. Monthly phone interview to assess the proper use of the app and provide motivation	In-person session to download and teach the app use during baseline visit. Measurement taken at baseline and 3 months; Nil further face-to-face interaction. Monthly phone calls (2 calls)	Received a diary to record food intake and exercise. Staff members provided health behavior change education at baseline visit; Health-related newsletters sent monthly, containing the same information about behavior as those received by the experimental group. Received monthly phone interview motivation and review	Weight change (kg), mean (SD) 3 months: I: –1.25 (1.14), *P*<.01, C: –0.42 (1.23), *P*<.07, between-groups *P*<.01; The effect was most pronounced in app users with good adherence (1.45 kg more weight reduction than the control group participants)
Lim et al [30] (2020), Singapore, multiracial RCT	Guided on the use of the app and educated on dietary and physical activity modification through a 1-hour face-to-face session with the research dietitian at the first study visit. Set individualized weight and lifestyle goals on the app. Advised to monitor lifestyle and progress on app. Educational videos and daily tips on healthy lifestyle available on app. Reminders and push notifications in place for meal and weight logging. Remote coaching with dietitians on progress, received encouragement and advice on app. Provided weighing scale	1-hour individual face-to-face education session with the research dietitian at baseline visit; Remote dietitian coaching on app; 2 optional workshops; Measurements at baseline, 3-month, and 6-month visits	Counseled individually for 30-40 minutes on diet and exercise by a nurse practitioner during baseline visit. Healthy food plate, physical activity, and the importance of weight loss are key areas of focus during the counseling. Provided weighing scale	Weight change (kg), mean (SD), 3 months: C: –0.8 (2.1), I: –3.2 (3.1), between-groups *P*<.001, 6 months: C: –0.5 (2.9), I: –3.2 (4.1), between-groups *P*<.001. achieved 5% weight loss, 3 months: C: 8%, I: 25%, 6 months: C: 8%, I: 44%
Muralidharan et al [31] (2019), India, Indian, multicenter RCT	Received app at baseline visit. App offered reality television show video lessons to educate and encourage lifestyle behavior tracking and change. Video lessons highlighted challenges and suitable solutions. Automatic motivational messages offered according to user's progress, alerts to prompt tracking, quizzes to reinforce learning, and message function to chat with coaches. Coaches provided weekly calls to revisit topics and emailed reports	Baseline visit to download app, weekly coach calls, emails, and text messages. Remote coach support through app chat. Measurements at baseline and 12-week visit	Received standard care that included a brochure on healthy eating, weight loss, and exercise at baseline visit. Offered face-to-face counseling with nutritionist	Weight change (kg), mean, 3 months: I: –1.1, within-group *P*<.01, C: –0.3, within-group *P*=.05, between-groups: –0.8, *P*<.05, achieved 5% weight loss: I: 15%, C: 9%
Oh et al [32] (2015), South Korea, Korean, multicenter RCT	Received information on increasing physical activity and controlling diet habits. Provided mobile phones for remote monitoring, body composition monitors, and pedometers. Advised to weigh daily or minimally 3 times weekly. Data were transmitted to the central server for immediate feedback through the designed algorithm. Received weekly, monthly health reports on progress through app; Provided phone consultations by educated consultants on disease management, health education, exercise, medication, and proper nutrition	Baseline visit, 12-week, and 24-week measurement visits. Remote app support and phone consultations by educated consultants	Received basic information on increasing physical activity and controlling diet habits at baseline visit. Body weight scales and pedometers were provided along with body weight journal for self-recording of weight and waist circumference; Returned for 2 in-person visits for measurements, consultations with physicians, and received advice about their nutrition and exercise	Weight change (kg), mean (SD) 6 months: I: –2.21 (3.60), *P*<.001, C: –0.77 (2.77), *P*<.001, between-groups *P*<.001
Shin et al [33] (2017), South Korea, Korean, pilot RCT	Received a 5-minute face-to-face education on diet and exercise from a nurse with standardized education material (1200 kcal sample menu), exercise recommendations, and behavior modification. Offered Fit.Life wireless physical activity tracker with Bluetooth transmission and detailed instructions on activity tracker use with demonstration and handouts. Provided clear activity goals and advised to track activity on app; Additional features for tracking progress to hit financial goals; Intervention I1: app, intervention I2: app+financial incentives	Baseline visit for 5-minute education, detailed demonstration, and instruction on app use; 4 measurement visits (baseline, week 4, week 8, and week 12)	Received a 5-minute face-to-face education on diet and exercise from a trained nurse. Content included the clinical consequence of obesity, a dietary recommendation for weight loss with an example of a 1200 kcal sample diet menu, and a physical activity recommendation with specification of frequency, intensity, time, and type	Weight change (kg), mean (SD) 3 months: I2 (app + financial incentives): –3.1 (3.7), I1 (app): –1.1 (2.9), C: –0.4 (2.5), *P* value between C and I2<.001, between C and I1=.38, and between I1 and I2=.006
Suen et al [34] (2019), Hong Kong, Chinese, feasibility RCT	Received coaching on applying auricular acupressure with instructions on frequency of application. Return demonstration was required to ensure proper treatment. Information booklet and mobile app provided. App provided daily reminders for self-pressing, encouraged compliance, and tracking of self-pressing and bowel movement; App provided relevant multimedia information and precaution on auricular acupressure. Users could communicate with researchers through app and were reminded of return visit dates on app	Researchers met patients twice weekly to change tapes. Remote communication and advice provided on app if patients had questions or problems	Received coaching on applying auricular acupressure with instructions on frequency of application. Return demonstration was required to ensure proper treatment; Information booklet provided. Patients were requested to manually record the frequency of daily pressing and bowel movement. Researchers met patients twice weekly to change tapes	Weight change (kg), mean, 8 weeks: C: –1.33, *P*=.005, I: –1.56, *P*<.001, no significant difference between groups
Tanaka et al [35] (2018), Japan, Japanese, RCT	Assigned to a group with up to 6 members where users could share meal photos in the group chat of the culturally tailored app, FiNC, and receive direct feedback, instructions, advice, and encouragement from a nutrition professional. Users could also communicate with other users for social support. Specific FiNC-method dietary recommendations were provided without any calorie restriction. Self-monitoring and group learning were encouraged on the app	Baseline, 8-week, and 12-week in-person measurements. No further human intervention. Remote communication with certified nutrition professional through app	Nil intervention provided during the 12-week waitlist period (Controls received intervention afterward for 8 weeks)	Weight change, mean (95% CI), 8 weeks: I: –1.4 (–2.0 to –0.8), C: –0.1 (–0.6 to 0.4), between-groups *P*=.001, week follow-up after active intervention, 12 weeks: I: –1.4 (–2.1 to –0.8), C: –0.1 (–0.7 to 0.6), between-groups *P*=.004
Yang et al [49] (2017), Taiwan, Chinese, crossover RCT	Received mobile physical activity promotion tool inclusive of lifestyle counseling, professional personal counseling, constructive feedback, health information, individualized reminder message at least once a week through Line app and email. A self-monitoring app with mobile activity sensor was provided along with an interactive internet webpage where users could track their health, compare results with peers, and receive recommendations	Lifestyle counseling at baseline, minimal human contact; Remote coaching on app and sending of reminder messages at least once weekly through Line and email; Baseline, 12-week, and 24-week in-person measurement visits	Received lifestyle counseling and booklet containing health education in support of behavioral and educational advice for diet control, increased physical activity, less smoking and drinking, stress, and regular health examination. Information on the related risk factors, development and prevention of metabolic syndrome, and various websites were also provided to the patients	Weight change (kg), mean (SD), 6 months, with activity promotion system: pre–activity promotion system: 77.7 (15.1), post–activity promotion system: 76.4 (15.5), pre–usual care: 78.1 (16.6), post–usual care: 76.8 (15.8), between-groups *P*=.93; (Crossover study without washout period, nil results on specific phases of intervention)
Yang et al [36] (2020), South Korea, Korean, cluster RCT	Physicians provided education on the use of the medical instruments and smartphone app. Explained management targets and guidelines to patients. Provided glucometer, test strips, and electronic manometer monitoring. Users were asked to upload their daily SMBG^f^ results, other biometric information, and weight through the app; Data automatically transmitted to the main server where physicians could check the results through a website and send feedback messages (praise, encouragement, feedback, and advice) at least once per week. Additional direct phone calls were conducted as required. Monthly face-to-face consultations offered	Baseline education on instruments, smartphone app, management targets and guidelines. Weekly feedback message through website or additional calls as necessary. Monthly in-person consultations to review progress, measurements, and management	Visited the private clinics and received face-to-face consultations every month for review and measurements	Weight change (kg), mean (95% CI), 3 months: I: −0.63 (−1.02 to −0.24), C: −0.88 (−2.65 to 0.90), adjusted mean difference to control: 0.22 (−1.26 to 1.71), between-groups *P*=.77
Zhang et al [37] (2019), China, Chinese, RCT	Group I1: app (basic), received basic diabetes education, including diet control, adequate exercise, SMBG, and regular review. Provided with glucose meter, test strips, and targets and encouraged to track BG^g^, habits, and obtain diabetes-related knowledge through the app. Users could contact clinicians by phone or app; Group I2: app, interactive group I1 intervention+third-party professional diabetes health care team comprising a dietitian and a health manager. Health team provided feedback and recommendations on progress, BG, and lifestyle habits. Provided daily prompts (first month) and then monthly on the app by health care team and reviewed weekly glucose reports. Users were given BG targets and were able to contact clinicians by phone or app	Baseline, 3-month, and 6-month measurement visits, ability to contact clinicians on the web through app or phone. Group I1 received support from a clinician, group I2 had additional interactive support on app with web-based management health care team comprising a dietitian and a health manager	Provided basic education. Patients obtained diabetes-related knowledge and skills by self-learning and summarizing, and they adopted lifestyles and behaviors voluntarily. Equipped with a designated BG meter and test strips, patients were advised to record results in a logbook. They could contact clinicians through phone	Weight change (kg), mean (SD), baseline: C: 69.6 (10.0), I1 (app, basic): 72.3 (11.6), I2 (app with health team): 70.8 (11.9), 3 months: C: 69.6 (9.6), I1: 72.2 (11.9), I2: 70.9 (11.6), 6 months: C: 69.4 (9.9), I1: 72.0 (11.7), I2: 71.0 (11.6); There were no significant differences among the 3 groups for body weight at both 3 and 6 months
Zhou et al [38] (2016), China, Chinese, pilot RCT	Downloaded the Welltang app at baseline visit and received diabetic knowledge on diet, exercise, medicine, blood glucose monitoring, and the latest guidelines for diabetes care. Users were asked to monitor 7-point finger blood glucose level for 1 day every 1-2 weeks (prompts in place) and track lifestyle habits. Advice on progress, values, target goals, and medication were offered by clinicians or study team through app once a week or fortnight. Users could communicate with clinicians through app, and an electronic action plan facilitated clinic review	Baseline, 1-month, 2-month, and 3-month in-person consultations. Remote interaction with clinicians on app as necessary. Weekly or fortnightly feedback from clinicians	Monthly visits to see physician to review blood glucose readings through logbooks. Patients were asked to monitor their 7-point finger capillary blood glucose level with a blood glucose meter 1-3 days before each clinic attendance to facilitate medication regimen adjustments	Weight change (kg), mean (SD), baseline: I: 62.4 (12.8), C: 62.5 (12.8), 3 months: I: 62.2 (11.0), C: 62.7 (12.1), *P* value not significant within or between groups
He et al [39] (2017), China, Chinese, cohort-based non-RCT	Received an official WeChat account for self-monitoring and immediate feedback on lifestyle habits. Users communicated and competed on weight loss progress. Users received scores for interactions, feedback information, or activity on the app, and top scorers were rewarded. Multimedia information on weight loss and an expert consulting group in place to answer questions	Nil baseline education or in-person session; 2 weight managers per work organization were trained to obtain participants’ data on height, weight, and waist circumference before and after the interventions were initiated for both groups. Remote communication with experts through app	Routine publicity, such as the slogan “Take the stairs and lose weight,” was provided to the control group. No further details specified	Weight change (kg), mean (SD): I: –2.09 (3.43), C: –1.78 (2.96), mean weight loss between the 2 groups for men was significant based on the stratification of age and educational level, weight loss changes were not significant for women
Kim et al [40] (2014), South Korea, Korean, matched controlled non-RCT	Baseline data recorded were transmitted to the app at first visit. Thereafter, users self-measured blood pressure and blood glucose levels, and data were automatically transmitted to hospital or medical staff through the app. Medical staff analyzed the data and sent tailored feedback to the patient once per week on average; App provided warning messages and advice when blood glucose levels were too high or too low. Study staff called users if they had hypoglycemia or no data were recorded	Nil baseline education. Measurement visits at baseline and 12 weeks. Remote supervision and weekly coaching on app. Staff called users if they had hypoglycemia or no data were recorded	Standard care, not clearly reported in paper	BMI change (kg/m^2^), mean (SD), baseline: I: 25.0 (3.3), C: 24.9 (3.4), 3 months: I: 25.0 (3.4), within-group *P*=.80, C: 24.3 (3.1), within-group *P*=.06, no significant difference between groups
Kim et al [50] (2019), South Korea, Korean, cohort-based non-RCT	Received face-to-face counseling service at public health center from physician, nutritionist, exercise specialist, and nurse at baseline, 12 weeks, and 24 weeks. Offered activity monitors, sphygmomanometers, glucometers, body composition measuring devices, and app for self-monitoring. Instructed to sync activity at least 5 times weekly and upload meal pictures once a month. Remote weekly individualized service related to healthy lifestyles was provided by health professionals along with monthly reports. Access to web-based communities for each health center facilitated consultations; Received intensive nutritional consultations at health centers based on meal photos (Rewards such as mobile gift cards were offered to users with excellent performances, but this was not duly reported in the *Methods* section)	Baseline, 12-week, and 24-week consultations and measurements. Weekly individualized advice and services related to lifestyle habits provided by physicians, nurses, nutritionists, and physical activity experts who monitored health information on the web in real time; Intensive nutrition consultations at each visit to health center	After classification according to test results, tailored care plans were established. Face-to-face counseling services offered at public health center by team comprising a health manager (a health expert such as a physician or nurse), nutritionist, and certified exercise expert, who provided individual or group health consultation (consultations adhered to the 2011 One-Stop Health Service Consultation Manual)	BMI measured at baseline but not reported in results. Change in proportion of patients with metabolic risk factor (elevated waist circumference) according to Adult Treatment Panel III criteria: I: –62 male patients, I: –78 female patients, significant difference within group for both genders, C: –2 male patients, C: –20 female patients, significant difference within female group only. No significant difference between groups
Wijaya and Widiantoro [41] (2018), Taiwan, Indonesian, pretest-posttest design	The intervention group received Social Cognitive Theory skill-building by WGTC^h^ for a 10-week program. The participants formed teams of 3 or 4 members based on friendship, received a booklet that provided physical activity–related knowledge, and were offered a 50-minute guidance on watch and app use at baseline. Individual and group performances were shown in the WGTC of the webpage where information was automatically transmitted by the iNCKU watches and the smartphone apps through daily use	Nil baseline education. Provision of items, booklet, guidance on use and purpose of study at baseline. Measurements at baseline and 10 weeks. No further human contact	Received explanation for the purposes of the study and a booklet that provided physical activity–related knowledge	Weight change (kg), mean (SD), baseline: I: 59.04 (65.44), C: 58.11 (66.83), 10 weeks: I: 57.78 (64.30), C: 57.92 (66.70), between-groups *P*=.64, intervention recorded lower body weight, *P*<.002

^a^RCT: randomized controlled trial.

^b^DPP: diabetes prevention program.

^c^I: intervention.

^d^C: control.

^e^SMART-CR/SP: Smartphone and Social Media–Based Cardiac Rehabilitation and Secondary Prevention.

^f^SMBG: self-monitoring blood glucose.

^g^BG: blood glucose.

^h^WGTC: web-based game with team competition.

The active intervention period ranged from 8 to 52 weeks, with a mean period of 18 weeks; the most common intervention periods were 6 months [25-28,30,32,37,39,49,50], followed by 3 months [29,31,33,35,36,38,40,47]. Of the 21 studies, only 1 study (5%) included a 4-week follow-up outcome measurement after the active intervention [35]. The average attrition rate was 11.5% (SD 8.53, range 0%-28%). Of the 21 studies, 2 (10%) consisted of single-component interventions using a mobile app exclusively [35,39], whereas 19 (90%) were multicomponent interventions incorporating additional components such as face-to-face consultations [25-30,32-34,36-38,47-50], reviews through phone calls or emails [26,27,29,31,32,36,40,48], supporting webpage [41], or financial incentives [33]. Of the 16 studies with face-to-face consultations, 4 (25%) also included tutorials to familiarize users on app use [29,30,36,41].

Across interventions, of the 21 studies, 12 (57%) used a researcher-designed app [27,28,30-34,36,40,41,49,50], whereas 9 (43%) used a commercially available app [25,26,29,35,37-39,47,48]. All studies had either used a culturally adapted app or had an app that was locally developed, except for 2 (10%) studies [29,47] that used a generic app developed in the United States. All studies reported using a multifunctional app, with the most common features being interactivity with health professionals; calorie, activity, or weight tracking; health and lifestyle information; and progress feedback (Multimedia Appendix 5 [25-41,47-50]). Other features such as gamification, which included scoring of app use to gain points for reward redemption and leader board positions, were less commonly reported. The average number of app features was 8.7 (SD 3.36, range 3-15). Comparing between commercially available and researcher-designed apps, the average numbers of app features were comparable at 7.7 (SD 3.15, range 3-12) and 9.5 (SD 3.45, range 5-15), respectively.

### Risk-of-Bias Assessments

#### RCTs and Bias

The risk-of-bias assessments of the included studies are summarized in Tables 3 and 4 for RCTs and Table 5 for non-RCTs, respectively. More than half of the RCTs included in both the systematic review and meta-analysis were rated low risk for selection bias, attrition bias, reporting bias, and other biases. Of the 17 RCTs, 1 (6%) was rated high risk for random sequence generation because of errors in patient randomization [27] and 5 (29%) were rated unclear risk for allocation concealment because of insufficient details reported by the authors [27-29,37,38]. Performance bias was rated high risk for 53% (9/17) of the studies [26-30,32,33,35,38], whereas 29% (5/17) of the studies scored unclear risk [25,31,34,36,37]. Of the 17 studies, detection bias was rated unclear for most, except for 3 (18%) that were rated low risk [25,34,35]. Of the 17 studies, 3 (18%) were rated high risk for attrition bias, with attrition rates between 21% and 24% [27,28,31]; 2 (12%) were rated unclear risk [32,38]; and 1 (6%) was rated high risk for reporting bias because the authors did not report the primary outcome registered in the trial registration [31]. Apart from these bias domains, of the 17 studies, 4 (24%) and 3 (18%) were rated high risk and unclear risk with regard to other biases, respectively.

**Table 3:** Risk of bias within the randomized controlled trials for selection, performance, and detection bias domains (N=17).

Author, year, country	Selection bias (random sequence generation)	Selection bias (allocation concealment)	Performance bias	Detection bias (self-reported outcomes)	Detection bias (objective measures)
Bender et al [47], 2018, United States	Low risk	Unclear risk	High risk	Low risk	Low risk
Dong et al [48], 2018, China	Low risk	Unclear risk	High risk	Unclear risk	Unclear risk
Dorje et al [25], 2019, China	Low risk	Low risk	Unclear risk	Unclear risk	Low risk
Kaur et al [26], 2020, India	Low risk	Low risk	High risk	Unclear risk	Unclear risk
Kim et al [27], 2019, South Korea	High risk	Unclear risk	High risk	Unclear risk	Unclear risk
Lee et al [28], 2018, South Korea	Low risk	Unclear risk	High risk	Unclear risk	Unclear risk
Lee et al [29], 2019, South Korea	Low risk	Unclear risk	High risk	High risk	Unclear risk
Lim et al [30], 2020, Singapore	Low risk	Low risk	High risk	Low risk	Unclear risk
Muralidharan et al [31], 2019, India	Low risk	Low risk	Unclear risk	Low risk	Unclear risk
Oh et al [32], 2015, South Korea	Low risk	Low risk	High risk	Unclear risk	Unclear risk
Shin et al [33], 2017, South Korea	Low risk	Low risk	High risk	Unclear risk	Unclear risk
Suen et al [34], 2019, Hong Kong	Low risk	Low risk	Unclear risk	Unclear risk	Low risk
Tanaka et al [35], 2018, Japan	Low risk	Low risk	High risk	Unclear risk	Low risk
Yang et al [49], 2017, Taiwan	Low risk	Unclear risk	High risk	Unclear risk	Unclear risk
Yang et al [36], 2020, South Korea	Low risk	Low risk	Unclear risk	Unclear risk	Unclear risk
Zhang et al [37], 2019, China	Low risk	Unclear risk	Unclear risk	Low risk	Unclear risk
Zhou et al [38], 2016, China	Low risk	Unclear risk	High risk	Unclear risk	Unclear risk

**Table 4:** Risk of bias within the randomized controlled trials for attrition, reporting, other, and overall bias domains (N=17).

Author, year, country	Attrition bias	Reporting bias	Other bias	Overall bias
Bender et al [47], 2018, United States	High risk	Low risk	High risk	High risk
Dong et al [48], 2018, China	Low risk	Low risk	Low risk	Moderate risk
Dorje et al [25], 2019, China	Low risk	Low risk	Low risk	Low risk
Kaur et al [26], 2020, India	Low risk	High risk	Unclear risk	High risk
Kim et al [27], 2019, South Korea	High risk	Low risk	High risk	High risk
Lee et al [28], 2018, South Korea	High risk	Low risk	High risk	High risk
Lee et al [29], 2019, South Korea	Low risk	Low Risk	Low risk	High risk
Lim et al [30], 2020, Singapore	Low risk	Low risk	Low risk	Moderate risk
Muralidharan et al [31], 2019, India	High risk	Unclear risk	High risk	High risk
Oh et al [32], 2015, South Korea	Unclear risk	Low risk	Unclear risk	Moderate risk
Shin et al [33], 2017, South Korea	Low risk	Low risk	Unclear risk	Moderate risk
Suen et al [34], 2019, Hong Kong	Low risk	Unclear risk	High risk	Moderate risk
Tanaka et al [35], 2018, Japan	Low risk	Low risk	Low risk	Moderate risk
Yang et al [49], 2017, Taiwan	High risk	Unclear risk	High risk	High risk
Yang et al [36], 2020, South Korea	Low risk	Unclear risk	Low risk	Moderate risk
Zhang et al [37], 2019, China	Low risk	Unclear risk	Low risk	Moderate risk
Zhou et al [38], 2016, China	Unclear risk	Low risk	Low risk	Moderate risk

**Table 5:** Risk of bias within the non-RCTs^a^ (N=4).

	Author, year, country			
	He et al [39], 2017, China	Kim et al [40], 2014, South Korea	Kim et al [50], 2019, South Korea	Wijaya and Widiantoro [41], 2018, Taiwan
Study design	Cohort-based non-RCT	Matched controlled non-RCT	Cohort based non-RCT	Pretest-posttest design
Bias due to confounding	Moderate risk	Serious risk	Critical risk	Moderate risk
Bias in selection of patients into the study	Low risk	Low risk	Low risk	Low risk
Bias in classification of interventions	Low risk	Low risk	Low risk	Low risk
Bias due to deviations from intended interventions	Moderate risk	Moderate risk	Serious risk	Low risk
Bias due to missing data	Low risk	Low risk	Low risk	Low risk
Bias in measurement of outcomes	Moderate risk	Serious risk	Moderate risk	Moderate risk
Bias in selection of the reported result	Moderate risk	Moderate risk	Moderate risk	Moderate risk
Overall bias	Moderate risk	Serious risk	Critical risk	Moderate risk

^a^RCT: randomized controlled trial.

#### Non-RCTs and Bias

The overall risk of bias for non-RCTs ranged from moderate to critical risk. All studies were rated low risk for selection bias, classification bias, and attrition bias. The studies were mostly rated moderate risk of bias for the other domains, except for 1 study that was rated serious risk for confounding bias and detection bias, leading to the overall rating of serious bias for the study [40]. A non-RCT that was excluded from the meta-analysis had an overall critical risk score because of critical risk of bias for confounding and serious risk of bias for deviation from the intended intervention [50].

### Intervention Efficacy

Of the 21 studies, 4 (19%; 3 RCTs and 1 non-RCT) were excluded from the meta-analysis because they did not provide specific values for weight-related outcomes that could be pooled [47-50]. In Bender et al [47], the authors reported significant weight loss and higher percentage of intervention patients achieving 5% weight loss, whereas the remaining 3 studies did not report any significant results.

Among the 14 selected RCTs for the quantitative analysis, 9 (64%) reported a positive effect for the intervention on weight loss between the treatment arms [26,28-33,35,47]. In the 8 RCTs that did not report significant differences between the treatment groups, 7 (88%) nonetheless found that the intervention arms contributed to greater weight loss compared with the controls [27,34,38,39,41,49,50]. In all, 3 RCTs assessed the proportion of participants achieving clinically significant weight loss (ie, 5% weight loss) and found that between 15% and 44% of the participants in the intervention groups achieved 5% weight loss, whereas 6%-9% of the control participants achieved clinically significant weight loss [30,31,47].

**Figure 2:**
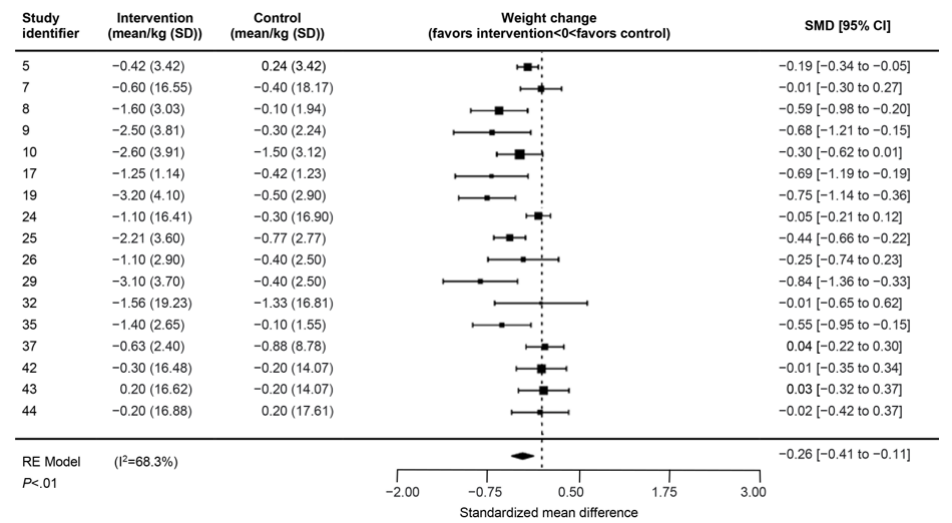
Forest plot showing the pooled effects of the interventions that incorporate apps on weight change. RE: random effects; SMD: standardized mean difference.

The pooled weighted effect size across 14 RCTs for weight change (Figure 2) was small to moderate (Hedges *g*=–0.26; 95% CI –0.41 to –0.11; *P*<.01) with substantial heterogeneity (I^2^=68.3%), whereas similar effect sizes were also observed for BMI (Hedges *g*=–0.21; 95% CI –0.42 to –0.01; *P*=.04; I^2^=69.9%) and waist circumference (Hedges *g*=–0.24; 95% CI –0.45 to –0.02; *P*=.03; I^2^=65.5%; Figures S1 and S2 of Multimedia Appendix 6). In terms of absolute weight, BMI, and waist circumference reduction, the raw mean difference revealed that the intervention group lost 1.16 kg (95% CI 0.81-1.52), 0.42 kg/m^2^ (95% CI 0.16-0.68), and 1.21 cm (95% CI 0.22-2.21) more than the control group, respectively (Figures S3-S5 of Multimedia Appendix 6). Visual assessment of the funnel plots revealed no obvious asymmetry, suggesting a low risk of publication bias (Multimedia Appendix 7).

In a separate analysis of non-RCTs, 2 studies were included for each outcome: weight and BMI (Figures S6 and S7 of Multimedia Appendix 6). The effect size for weight change was statistically significant but small, with Hedges *g*=–0.09 (95% CI –0.13 to –0.05; *P*<.01; I^2^=0%). In contrast, the effect size for BMI was not statistically significant (Hedges *g*=0.06; 95% CI –0.27 to 0.39; *P*=.74; I^2^=0%). No analysis was conducted for waist circumference because only 1 study reported this outcome [39]. Overall, the results for the non-RCT meta-analysis should be interpreted with caution because there were very few data points included in the analyses and the data from the studies included were highly variable.

In the subgroup analyses for single-component (standalone app interventions) studies, no meta-analysis was conducted because of a lack of data points to assess the outcomes for RCTs and non-RCTs separately. Tanaka et al [35] reported a statistically significant weight loss between the groups but not He et al [39], although the intervention patients achieved a greater weight loss.

**Figure 3:**
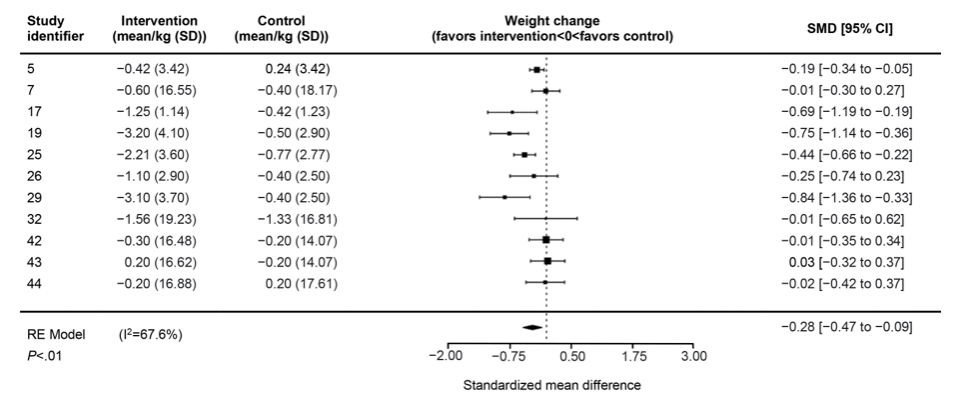
Forest plot showing the pooled effects of interventions combining usual care with app (intervention) versus usual care alone (control) on weight change. RE: random effects; SMD: standardized mean difference.

For interventions with the addition of apps to usual care, the effect size for weight (Figure 3) was statistically significant with a small to moderate Hedges *g*=–0.28 (95% CI –0.47 to –0.09; *P*<.01; I^2^=67.6%); however, this was not the case for BMI and waist circumference outcomes (Figures S8 and S9 of Multimedia Appendix 6).

Moderation analysis suggested that study duration was not a significant moderator of intervention effects on weight, BMI, and waist circumference, with *P* values of .72, .67, and .69, respectively. All studies included in this meta-analysis had an intervention period of 6 months or less. Post hoc analyses (Figures S10 and S11 of Multimedia Appendix 6) revealed that changes in body weight were significantly different between the intervention and control groups, both in studies with a duration of 3 months or less (Hedges *g*=–0.28; 95% CI –0.52 to –0.05; *P*=.02; I^2^=75%), as well as those between 3 and 6 months (Hedges *g*=–0.29; 95% CI –0.51 to –0.08; *P*=.01; I^2^=72.2%). Conversely, changes in BMI and waist circumference did not differ significantly between the treatment arms in both data subsets (Figures S12-S15 of Multimedia Appendix 6).

### Secondary Outcomes

Across all 21 studies, 17 (81%) reported at least one secondary outcome measured through a range of tools and approaches, as described in Multimedia Appendix 8 [25-41,47-50]. In all, 8 out of 11 studies (73%) showed that an app intervention improved dietary outcomes, with 5 studies [26,32,38,48,50] reporting concomitant within-group and between-groups differences, whereas Suen et al [34] and Lee et al [29] reported within-group differences. Physical activity outcomes were mostly reported as changes in exercise frequency, intensity, or duration. A total of 7 out of 11 studies (64%) showed an increase in physical activity level or exercise scores, with significant within-group and between-groups differences reported in 3 [29,38,50] and 6 studies [25,33,38,41,49,50], respectively. Significant improvements in self-efficacy scores were reported in 4 out of 6 studies (67%) [26,38,48,50], whereas 2 studies reported no significant change [27,41].

Among the 9 studies reporting significant weight change, 2 (22%) [26,32] reported significant improvements in diet, whereas 1 (11%) [33] reported significant increment in physical activity. Of the 9 studies reporting significant weight change, 3 (33%) studies did not find a significant improvement in diet [29,33,35], whereas the others did not report dietary outcomes [28,30,31,47]. Similarly, for physical activity, 3 (33%) studies [28,29,32] did not find any significant improvement, whereas the remaining studies did not report this outcome [26,30,31,35,47].

Of the 21 studies, 9 (43%) reported on app use and engagement [25,29,30,33,35,37-39,50]; however, only 6 offered use statistics, albeit by using various measures [25,30,35,37-39]. These statistics included number of daily log-ins [30]; meal, activity, or weight logging [30]; uploading of meal photos [35]; reading of messages [25]; frequency of app use [37]; interactivity with coaches [37,38]; and number of questions asked and cumulative app use scores [39]. Of the 9 studies, 4 (44%) reported that increased app use or adherence was associated with greater weight loss and health outcomes [29,33,35,39], whereas the remaining 5 (56%) did not explore any associations. In addition, of the 9 studies, 2 (22%) found that app use declined over time [30,35], whereas the remaining 7 (78%) did not report app engagement trends.

## Discussion

### Principal Findings

The interventions that incorporated apps produced a small to moderate effect in reducing weight, BMI, and waist circumference in Asian populations, although substantial heterogeneity was present. It was unclear if single-component standalone app studies were efficacious for weight loss; however, supplementing usual care with an app seemed to be beneficial for enhancing weight loss compared with usual care alone. However, the results may not be representative of long-term studies because of a lack of data. This review also found that app interventions may be beneficial for improving diet and increasing physical activity and self-efficacy for healthy behaviors. In these interventions, apps made for Asian populations were largely culturally adapted and multifunctional, with the most common app features being communication with health professionals and self-monitoring of behaviors and outcomes. Overall, the quality of the studies ranged from low to unclear risk of bias for most domains, apart from performance bias where most of the studies were graded high risk because of the lack of blinding, which is challenging in interventions that incorporate apps. Therefore, the results should be interpreted with caution.

Among the studies in our review that reported a significant difference in either between groups or within group for weight loss favoring the intervention or a greater likelihood among the intervention groups for clinically significant weight loss, most were multicomponent. They typically included face-to-face consultations and reviews through phone calls or emails in addition to the app component.

Our review found that supplementing multicomponent usual care practices with an app was successful in achieving greater results. Reviews of studies in Western populations have observed similar findings [8,15]. This could be attributed to the provision of social support, accountability, and increased opportunities for patients to be reviewed beyond the confines of the app [51], thus underscoring the importance of additional components to raise contact frequency, enhance self-monitoring, and maximize outcomes.

Monitoring of weight, diet, and physical activity behaviors was a common feature of apps in the interventions reviewed. The addition of an app to usual care aligns with the understanding that self-monitoring can improve self-regulation of behaviors and weight loss [52,53]. By enhancing convenience for users, apps thus encourage more consistent self-monitoring [54] to promote treatment adherence and weight loss [15,42]. This review also observed that the outcomes of healthy eating and increased physical activity, which are key determinants of weight loss, tend to occur alongside improved self-efficacy for implementing healthy behaviors. As self-efficacy was previously found to be positively associated with self-monitoring, it is likely that increased self-monitoring may account for the behavioral improvements seen [55].

It was also apparent from our review that multifunctional, *all-in-one* apps were common among Asian interventions. The features included direct communication with health professionals through the app, in addition to functionalities for calorie, activity, and weight tracking as well as provision of health information and progress feedback, thereby matching the features found in effective digital weight loss interventions reported in a recent review [54].

In contrast with apps designed in Western countries, which tend to focus more on independent learning [8], apps designed for Asians frequently include accessibility to health professionals. It is plausible that having health coaches within apps reduces the barriers for Asians to seek health information, validation, and support from their clinicians [18,19,56], while conferring increased credibility to the coaching and support given to users [54,57]. App users recognize the benefit of health professionals such as dietitians providing support, particularly as they offer effective, evidence-based, culturally appropriate, and tailored dietary counseling to participants [58,59]. Correspondingly, most studies that employed a dietitian or nutrition-trained professional reported a greater weight loss with the intervention [28,30-32,35,37,50].

Furthermore, all but 2 studies [29,47] either used a culturally adapted app or an app that was locally developed; employed the respective country’s native language; and incorporated localized educational content, food databases, and recommendations. Research supports that using culturally appropriate content, engaging local facilitators, and offering the app in the native language are important factors that may promote app use and outcomes [57,60].

Only 6 of the 21 studies reported app use statistics, whereas 2 others provided associations between app adherence and outcomes without reporting actual app engagement data. Meyerowitz-Katz et al [61] reported in a recent meta-analysis that the pooled estimate of app nonuse (defined as attrition rate) was 43%, indicating a serious limitation of app-based interventions if strategies for maintaining long-term engagement with the intervention (longer than a year) are not considered. In this review, the interventions that incorporated an app achieved statistical significance for weight change in studies with durations that were 6 months or less. However, the efficacy of these interventions in long-term studies remains unclear because none of the studies reviewed included durations longer than a year. App engagement levels in this review varied across studies, making comparison difficult. Nevertheless, evidence from this review echoed the results reported by previous reviews that increased app use is associated with greater adherence and weight loss [8,15], notwithstanding the fact that app engagement typically declines over time [8,61].

### Strengths and Limitations

This is the first meta-analysis to report on the efficacy of apps incorporated into interventions targeting weight loss with or without healthy behavioral change in populations of Asian ethnicity. The review and meta-analysis were conducted according to best practice and followed PRISMA guidelines with a comprehensive search strategy and assessment of risk of bias using Cochrane Collaboration tools. The study selection, data extraction, and quality assessment were conducted independently by 2 reviewers.

This review is not without some limitations. The heterogeneity observed across studies was substantial, making it challenging to effectively interpret the results. Substantial heterogeneity could be due to the differences in study aims, targeted outcomes, methods, populations, and interventions. The lack of consistent and detailed reporting among the studies limited our ability to assess the true dose of intervention received, user engagement levels, and behavior change techniques that may have been employed in the apps. Therefore, the results of the meta-analysis should be interpreted with caution. Future studies that incorporate apps should consider using a standardized tool such as the Behavior Change Technique Taxonomy to code app features in a systematic and replicable manner and report user engagement statistics to evaluate app use and outcomes. This review was also limited to studies that were published in the English language. This may be problematic, given that we were studying apps in Asian populations, and English may not have been the first language in many countries; hence, some articles in other languages would not have been captured in this review. Finally, as most of the studies were multicomponent in nature, components apart from the app, such as in-person education or review calls, may have more strongly influenced the outcomes; however, it was not possible to identify the contribution of these components to the weight loss outcomes.

### Conclusions

This review contributes to the literature by presenting quantitative evidence that multicomponent interventions that incorporate apps produce a small to moderate effect toward weight loss in studies of Asian populations with intervention periods of 6 months or less. It is unclear if single-component standalone-app studies are efficacious for weight loss; however, adding apps to multicomponent usual care confers better outcomes. More evidence is required to determine the efficacy of apps in the long term. Cultural adaptation and offering the app in the native language of the users seem to be a priority in Asian apps. Multifunctional apps with features that include self-monitoring, individualized feedback, and a link to health professionals within the apps may be key to raising awareness and engagement, as well as promoting weight loss. Finally, it is recommended that investigators monitor and address the low uptake of apps and attempt to enhance engagement level before using apps as part of national health strategies for reducing obesity.

## Appendix

Multimedia Appendix 1 Modifications to PROSPERO protocol.

Multimedia Appendix 2 PRISMA (Preferred Reporting Items for Systematic Reviews and Meta-Analyses) checklist.

Multimedia Appendix 3 Search strategy for MEDLINE.

Multimedia Appendix 4 Unique identifiers for meta-analyses.

Multimedia Appendix 5 App features.

Multimedia Appendix 6 Additional meta-analysis and subgroup analyses.

Multimedia Appendix 7 Funnel plots of meta-analysis.

Multimedia Appendix 8 Secondary outcomes and app engagement data.

## Abbreviations

RCT: randomized controlled trial
