# Supplementary material for: Efficacy of Interventions That Incorporate Mobile Apps in Facilitating Weight Loss and Health Behavior Change in the Asian Population: Systematic Review and Meta-analysis
Source: J Med Internet Res. 2021 Nov 16;23(11):e28185. doi: 10.2196/28185 (PMC8663646; doi:10.2196/28185)
Supplement: Multimedia Appendix 1 [file jmir_v23i11e28185_app1.pdf]

Modifications to PROSPERO

We made the following modifications and provided an explanation for each change:

1. Edited the title of the review from "Efficacy of mobile application in facilitating weight loss and health behavior change in the Asian population: a systematic review and meta-analysis" to "Efficacy of interventions incorporating mobile applications in facilitating weight loss and health behavior change in the Asian population: a systematic review and meta-analysis" to more accurately reflect the type of intervention that was assessed in the review.
2. Added more authors to the review along with updated author information to provide statistical support and medical interpretation for the review.
3. Added an additional database, Global Health, post discussion with the librarian to ensure we were able to adequately capture the existing literature of interest.
4. We removed sleep as an additional outcome as none of the studies included sleep as a measured outcome.
5. The GRADE approach to evaluate the body of evidence was considered but the nature of the review question and the number and nature of the outcomes lead us to discard that approach to assess the validity of the body of evidence. Instead, we focused on using the Cochrane Collaboration Risk of Bias Tool and ROBINS-I tool to assess the risk of bias in studies.
