# Supplementary material for: Efficacy of Interventions That Incorporate Mobile Apps in Facilitating Weight Loss and Health Behavior Change in the Asian Population: Systematic Review and Meta-analysis
Source: J Med Internet Res. 2021 Nov 16;23(11):e28185. doi: 10.2196/28185 (PMC8663646; doi:10.2196/28185)
Supplement: Multimedia Appendix 3 [file jmir_v23i11e28185_app3.pdf]

Figure S1: Sample search strategy on MEDLINE

Medline 1.6.20 n=378

#	Searches	Results	Type	Actions	Annotations
1	Asia/	28037	Advanced	Display Results More	
2	exp Asia, Southeastern/	93980	Advanced	Display Results More	
3	Asia, Western/	1931	Advanced	Display Results More	
4	exp Far East/	401669	Advanced	Display Results More	
5	exp India/	101965	Advanced	Display Results More	
6	Bangladesh/	10797	Advanced	Display Results More	
7	Sri Lanka/	5946	Advanced	Display Results More	
8	(Asia* or China or Chinese or Korea* or Japan* or India* or Bangladesh* or Sri Lanka* or Vietnam* or Taiwan* or Philippin* or Filipin* or Malay* or Indonesia* or Thai* or Hong Kong or Singapore* or Cambodia*).mp,cp,in.	4331083	Advanced	Display Results More	
9	or/1-8	4334846	Advanced	Display Results More	
10	Mobile applications/	5682	Advanced	Display Results	

Figure S1 (continue)

			More
11	exp Cell Phone/	10534	Advanced Display Results More
12	exp Computers, Handheld/	7687	Advanced Display Results More
13	Telemedicine/	22145	Advanced Display Results More
14	Fitness trackers/	507	Advanced Display Results More
15	Wearable electronic devices/	2310	Advanced Display Results More
16	(app or apps).mp.	21718	Advanced Display Results More
17	Mobile*.mp.	82172	Advanced Display Results More
18	(Hand held or handheld).mp.	13109	Advanced Display Results More
19	(Smartphone* or smart phone*).mp.	8150	Advanced Display Results More
20	(Cell* phone* or cellphone*).mp.	9851	Advanced Display Results More
21	(mHealth or m-Health or mobile health).mp.	7507	Advanced Display Results More
22	Telemedicine.mp.	24745	Advanced Display

Figure S1 (continue)

				Results More
☐	23	Fitbit.mp.	304	Advanced Display Results More
☐	24	(Fitness tracker* or activity tracker*).mp.	676	Advanced Display Results More
☐	25	(Wearable adj5 device*).mp.	3743	Advanced Display Results More
☐	26	(Smart watch* or smartwatch*).mp.	217	Advanced Display Results More
☐	27	or/10-26	143294	Advanced Display Results More
☐	28	Weight loss/	36036	Advanced Display Results More
☐	29	Overweight/	24453	Advanced Display Results More
☐	30	Obesity/	178734	Advanced Display Results More
☐	31	Obesity management/	106	Advanced Display Results More
☐	32	Body mass index/	125399	Advanced Display Results More
☐	33	Body weight changes/	5	Advanced Display Results More

Figure S1 (continue)

☐	34	Waist circumference/	10219	Advanced Display Results More
☐	35	(Weight adj3 (loss* or los* or change* or variation or reduc* or differen* or maintain* or maintenance or control or management)).mp.	152723	Advanced Display Results More
☐	36	Waist circumference.mp.	24311	Advanced Display Results More
☐	37	(Body mass index or BMI).mp.	242323	Advanced Display Results More
☐	38	Obes*.mp.	307200	Advanced Display Results More
☐	39	Overweight.mp.	62436	Advanced Display Results More
☐	40	Body composition.mp.	52406	Advanced Display Results More
☐	41	or/28-40	585587	Advanced Display Results More
☐	42	9 and 27 and 41	425	Advanced Display Results More
☐	43	limit 42 to yr="2008 -Current"	378	Advanced Display Results More
