# Supplementary material for: Efficacy of Interventions That Incorporate Mobile Apps in Facilitating Weight Loss and Health Behavior Change in the Asian Population: Systematic Review and Meta-analysis
Source: J Med Internet Res. 2021 Nov 16;23(11):e28185. doi: 10.2196/28185 (PMC8663646; doi:10.2196/28185)
Supplement: Multimedia Appendix 4 [file jmir_v23i11e28185_app4.pdf]

Study Identifier	Author	Study Type (RCT/ non-RCT)	Mean Change (Intervention)	Standard Deviation (Intervention)	Mean Change (Control)	Standard Deviation (Control)	Sample number (Intervention)	Sample number (Control)	Outcome Measure	Study Period (months)	Subgroup	Study Aims	Single/ Multi-component	Units	Control Treatment	Intervention Treatment
1	Dorje et al. 2019	RCT	-0.5	4.17253	-0.2	4.74236	156	156	BMI	2	Not Applicable	Alternative focus	Multi	kg/m2	Usual Care	Usual care + App
2	Dorje et al. 2019	RCT	-0.6	4.60977	-0.9	4.74236	156	156	BMI	6	Not Applicable	Alternative focus	Multi	kg/m2	Usual Care	Usual care + App
3	Dorje et al. 2019	RCT	0	0.14142	0	0.10000	156	156	Waist circumference	2	Not Applicable	Alternative focus	Multi	cm	Usual Care	Usual care + App
4	Dorje et al. 2019	RCT	0	0.10000	0	0.14142	156	156	Waist circumference	6	Not Applicable	Alternative focus	Multi	cm	Usual Care	Usual care + App
5	Kaur et al. 2020	RCT	-0.42	3.41627	0.24	3.41627	366	366	Weight	6	Not Applicable	Alternative focus	Multi	kg	Usual + Lifestyle education	Usual + Lifestyle education + App
6	Kaur et al. 2020	RCT	-0.16	1.31770	0.09	1.12248	366	366	BMI	6	Not Applicable	Alternative focus	Multi	kg/m2	Usual + Lifestyle education	Usual + Lifestyle education + App
7	Kim et al. 2019	RCT	-0.6	16.54690	-0.4	18.17388	97	94	Weight	6	Not Applicable	Alternative focus	Multi	kg	Usual + Lifestyle education	Usual + Lifestyle education + App
8	Lee et al. 2018	RCT	-1.6	3.03000	-0.1	1.94000	52	53	Weight	6	Insufficiently active	Alternative focus	Multi	kg	Usual Care	Usual care + App + follow-up reviews
9	Lee et al. 2018	RCT	-2.5	3.81000	-0.3	2.24000	31	27	Weight	6	Minimally active	Alternative focus	Multi	kg	Usual Care	Usual care + App + follow-up reviews
10	Lee et al. 2018	RCT	-2.6	3.91000	-1.5	3.12000	94	67	Weight	6	Health-enhancing physical activity	Alternative focus	Multi	kg	Usual Care	Usual care + App + follow-up reviews
11	Lee et al. 2018	RCT	-0.7	1.14000	-0.2	0.93000	52	53	BMI	6	Insufficiently active	Alternative focus	Multi	kg/m2	Usual Care	Usual care + App + follow-up reviews
12	Lee et al. 2018	RCT	-0.9	1.30000	-0.2	0.86000	31	27	BMI	6	Minimally active	Alternative focus	Multi	kg/m2	Usual Care	Usual care + App + follow-up reviews
13	Lee et al. 2018	RCT	-1	1.44000	-1.3	6.70000	94	67	BMI	6	Health-enhancing physical activity	Alternative focus	Multi	kg/m2	Usual Care	Usual care + App + follow-up reviews
14	Lee et al. 2018	RCT	10.9	103.88000	0	13.24000	52	53	Waist circumference	6	Insufficiently active	Alternative focus	Multi	cm	Usual Care	Usual care + App + follow-up reviews
15	Lee et al. 2018	RCT	-3.8	5.78000	-0.5	2.93000	31	27	Waist circumference	6	Minimally active	Alternative focus	Multi	cm	Usual Care	Usual care + App + follow-up reviews
16	Lee et al. 2018	RCT	-4.4	4.75000	-3	4.08000	94	67	Waist circumference	6	Health-enhancing physical activity	Alternative focus	Multi	cm	Usual Care	Usual care + App + follow-up reviews
17	Lee et al. 2019	RCT	-1.25	1.14000	-0.42	1.23000	32	33	Weight	3	Not Applicable	Weight focus	Multi	kg	Usual + Lifestyle education	Usual + Lifestyle education + App
18	Lim et al. 2020	RCT	-3.2	3.10000	-0.8	2.10000	55	53	Weight	3	Not Applicable	Weight focus	Multi	kg	Usual + Lifestyle education	Usual + Lifestyle education + App
19	Lim et al. 2020	RCT	-3.2	4.10000	-0.5	2.90000	55	53	Weight	6	Not Applicable	Weight focus	Multi	kg	Usual + Lifestyle education	Usual + Lifestyle education + App
20	Lim et al. 2020	RCT	-1.3	1.10000	-0.4	0.80000	55	53	BMI	3	Not Applicable	Weight focus	Multi	kg/m2	Usual + Lifestyle education	Usual + Lifestyle education + App
21	Lim et al. 2020	RCT	-1.3	1.40000	-0.3	1.10000	55	53	BMI	6	Not Applicable	Weight focus	Multi	kg/m2	Usual + Lifestyle education	Usual + Lifestyle education + App
22	Lim et al. 2020	RCT	-3.4	5.10000	0.4	4.70000	55	53	Waist circumference	3	Not Applicable	Weight focus	Multi	cm	Usual + Lifestyle education	Usual + Lifestyle education + App
23	Lim et al. 2020	RCT	-2.9	5.00000	0.7	4.40000	55	53	Waist circumference	6	Not Applicable	Weight focus	Multi	cm	Usual + Lifestyle education	Usual + Lifestyle education + App
24	Muralidharan et al. 2019	RCT	-1.1	16.40731	-0.3	16.90118	271	290	Weight	3	Not Applicable	Weight focus	Multi	kg	Usual + Lifestyle education	Usual + Lifestyle education + App + weekly call follow up
25	Oh et al. 2015	RCT	-2.21	3.60000	-0.77	2.77000	181	153	Weight	6	Not Applicable	Weight focus	Multi	kg	Usual + Lifestyle education	Usual + Lifestyle education + App
26	Shin et al. 2017	RCT	-1.1	2.90000	-0.4	2.50000	34	32	Weight	3	App	Weight focus	Multi	kg	Usual + Lifestyle education	Usual + Lifestyle education + App
27	Shin et al. 2017	RCT	-0.5	0.80000	-0.2	0.70000	34	32	BMI	3	App	Weight focus	Multi	kg/m2	Usual + Lifestyle education	Usual + Lifestyle education + App
28	Shin et al. 2017	RCT	-2.4	2.30000	-1.2	3.00000	34	32	Waist circumference	3	App	Weight focus	Multi	cm	Usual + Lifestyle education	Usual + Lifestyle education + App
29	Shin et al. 2017	RCT	-3.1	3.70000	-0.4	2.50000	32	32	Weight	3	App with incentives	Weight focus	Multi	kg	Usual + Lifestyle education	Usual + Lifestyle education + App
30	Shin et al. 2017	RCT	-1	1.20000	-0.2	0.70000	32	32	BMI	3	App with incentives	Weight focus	Multi	kg/m2	Usual + Lifestyle education	Usual + Lifestyle education + App
31	Shin et al. 2017	RCT	-4.4	3.30000	-1.2	3.00000	32	32	Waist circumference	3	App with incentives	Weight focus	Multi	cm	Usual + Lifestyle education	Usual + Lifestyle education + App
32	Suen et al. 2019	RCT	-1.56	19.22718	-1.33	16.81135	19	19	Weight	2	Not Applicable	Weight focus	Multi	kg	Usual Care	Usual care + App
33	Suen et al. 2019	RCT	-0.6	10.97769	-0.5	6.22525	19	19	BMI	2	Not Applicable	Weight focus	Multi	kg/m2	Usual Care	Usual care + App
34	Suen et al. 2019	RCT	-1.6	15.62025	-2.81	15.18158	19	19	Waist circumference	2	Not Applicable	Weight focus	Multi	cm	Usual Care	Usual care + App
35	Tanaka et al. 2018	RCT	-1.4	2.65109	-0.1	1.55172	75	37	Weight	2	Not Applicable	Weight focus	Single	kg	No treatment	App
36	Tanaka et al. 2018	RCT	-1.9	3.97664	-0.9	3.56896	75	37	Waist circumference	2	Not Applicable	Weight focus	Single	cm	No treatment	App
37	Yang et al. 2020	RCT	-0.63	2.39603	-0.88	8.78023	145	94	Weight	3	Not Applicable	Alternative focus	Multi	kg	Usual Care	Usual care + App + direct calls as required
38	Yang et al. 2020	RCT	-0.26	0.89083	-0.41	3.98202	145	94	BMI	3	Not Applicable	Alternative focus	Multi	kg/m2	Usual Care	Usual care + App + direct calls as required
39	Yang et al. 2020	RCT	-0.93	3.25614	-0.88	3.58629	145	94	Waist circumference	3	Not Applicable	Alternative focus	Multi	cm	Usual Care	Usual care + App + direct calls as required
40	Zhang et al. 2019	RCT	-0.1	16.61836	0	13.86217	67	63	Weight	3	App with physician	Alternative focus	Multi	kg	Usual + Lifestyle education	Usual + Lifestyle education + App
41	Zhang et al. 2019	RCT	0.1	16.61836	0	13.86217	64	63	Weight	3	App with interactive team	Alternative focus	Multi	kg	Usual + Lifestyle education	Usual + Lifestyle education + App
42	Zhang et al. 2019	RCT	-0.3	16.47573	-0.2	14.07160	67	63	Weight	6	App with physician	Alternative focus	Multi	kg	Usual + Lifestyle education	Usual + Lifestyle education + App
43	Zhang et al. 2019	RCT	0.2	16.61836	-0.2	14.07160	64	63	Weight	6	App with interactive team	Alternative focus	Multi	kg	Usual + Lifestyle education	Usual + Lifestyle education + App
44	Zhou et al. 2016	RCT	-0.2	16.87720	0.2	17.61391	50	50	Weight	3	Not Applicable	Alternative focus	Multi	kg	Usual Care	Usual care + App
45	Zhou et al. 2016	RCT	-0.3	5.43548	0.09	5.55317	50	50	BMI	3	Not Applicable	Alternative focus	Multi	kg/m2	Usual Care	Usual care + App
46	Zhou et al. 2016	RCT	0	14.28635	0	14.70850	50	50	Waist circumference	3	Not Applicable	Alternative focus	Multi	cm	Usual Care	Usual care + App
47	He et al. 2017	non-RCT	-2.09	3.43000	-1.78	2.96000	11843	3467	Weight	6	Not Applicable	Weight focus	Single	kg	No treatment	App
48	He et al. 2017	non-RCT	-2.74	4.48000	-2.39	3.91000	11843	3467	Waist circumference	6	Not Applicable	Weight focus	Single	cm	No treatment	App
49	Kim et al. 2014	non-RCT	0	4.73814	-0.6	4.60108	35	35	BMI	3	Not Applicable	Alternative focus	Multi	kg/m2	No treatment	App + follow-up calls as required
50	Wijaya et al. 2018	non-RCT	-1.26	91.74357	-0.19	94.42001	34	36	Weight	2.5	Not Applicable	Alternative focus	Multi	kg	Usual Care	Usual care + App
51	Wijaya et al. 2018	non-RCT	-0.49	33.74484	-0.05	34.57046	34	36	BMI	2.5	Not Applicable	Alternative focus	Multi	kg/m2	Usual Care	Usual care + App
