# Supplementary material for: Efficacy of Interventions That Incorporate Mobile Apps in Facilitating Weight Loss and Health Behavior Change in the Asian Population: Systematic Review and Meta-analysis
Source: J Med Internet Res. 2021 Nov 16;23(11):e28185. doi: 10.2196/28185 (PMC8663646; doi:10.2196/28185)
Supplement: Multimedia Appendix 5 [file jmir_v23i11e28185_app5.pdf]

Table S3: App features incorporated in the respective apps utilised in studies included in the review

Author (Year), Country, Ethnicity	Weight-related healthy lifestyle information	Disease-related health information	Multimedia education materials	Guided advice for lifestyle behaviours	Weight goal setting	Diet goal setting	Activity goal setting
**Bender et. al.[30] (2018), United States, Filipino American**							
**Dong et. al.[31] (2018), China, Chinese**	Yes	Yes	Yes				
**Dorje et. al.[32] (2019), China, Chinese**	Yes	Yes	Yes	Yes			Yes
**Kaur et. al.[33] (2020), India, Indian**	Yes		Yes				
**Kim et. al.[34] (2019), South Korea, Korean**	Yes	Yes	Yes			Yes	Yes
**Lee et. al.[35] (2018), South Korea, Koreans**	Yes		Yes				
**Lee et. al.[36] (2019), South Korea, Koreans**	Yes			Yes	Yes		
**Lim et. al.[37] (2020), Singapore, Multi-racial**	Yes		Yes	Yes	Yes	Yes	Yes
**Muralidharan et. al.[38] (2019), India, Indian**	Yes		Yes	Yes	Yes	Yes	Yes
**Oh et. al.[39] (2015), South Korea, Koreans**	Yes	Yes		Yes	Yes		
**Shin et. al.[40] (2017), South Korea, Korean**							Yes
**Suen et. al.[41] (2019), Hong Kong, Chinese**	Yes		Yes	Yes			
**Tanaka et. al.[42] (2018), Japan, Japanese**	Yes			Yes			
**Yang et. al.[43] (2017), Taiwan, Chinese**	Yes						
**Yang et. al.[44] (2020), South Korea, Korean**		Yes					
**Zhang et. al.[45] (2019), China, Chinese**		Yes		Yes (for group C)			
**Zhou et. al.[46] (2016), China, Chinese**		Yes					
**He et. al.[47] (2017), China, Chinese**	Yes		Yes				
**Kim et. al.[48] (2014), South Korea, Korean**		Yes					
**Kim et. al.[49] (2019), South Korea, Korean**	Yes			Yes			
**Wijaya and Widiantoro[50] (2018), Taiwan, Indonesian**							Yes
**No. of studies with feature**	14	8	9	9	4	3	6

Table S3 (continue)

Author (Year), Country, Ethnicity	Disease-related outcome goal setting	Weight tracking/ self-monitoring	Diet tracking/ self-monitoring	Activity tracking/ self-monitoring	Tracking of other health variables/ behaviours	Integration of monitoring device to app	Automated feedback
**Bender et. al.[30] (2018), United States, Filipino American**		Yes	Yes	Yes		Yes	
**Dong et. al.[31] (2018), China, Chinese**							
**Dorje et. al.[32] (2019), China, Chinese**				Yes	Yes	Yes	
**Kaur et. al.[33] (2020), India, Indian**							
**Kim et. al.[34] (2019), South Korea, Korean**	Yes		Yes	Yes	Yes	Yes	Yes
**Lee et. al.[35] (2018), South Korea, Koreans**		Yes				Yes	Yes
**Lee et. al.[36] (2019), South Korea, Koreans**		Yes	Yes	Yes			Yes
**Lim et. al.[37] (2020), Singapore, Multi-racial**		Yes	Yes	Yes			Yes
**Muralidharan et. al.[38] (2019), India, Indian**		Yes	Yes	Yes			Yes
**Oh et. al.[39] (2015), South Korea, Koreans**		Yes		Yes	Yes	Yes	Yes
**Shin et. al.[40] (2017), South Korea, Korean**				Yes		Yes	Yes
**Suen et. al.[41] (2019), Hong Kong, Chinese**					Yes		Yes
**Tanaka et. al.[42] (2018), Japan, Japanese**		Yes	Yes				
**Yang et. al.[43] (2017), Taiwan, Chinese**			Yes	Yes		Yes	
**Yang et. al.[44] (2020), South Korea, Korean**	Yes	Yes			Yes	Yes	
**Zhang et. al.[45] (2019), China, Chinese**	Yes	Yes	Yes	Yes	Yes		
**Zhou et. al.[46] (2016), China, Chinese**	Yes	Yes	Yes	Yes	Yes		
**He et. al.[47] (2017), China, Chinese**		Yes	Yes	Yes			Yes
**Kim et. al.[48] (2014), South Korea, Korean**	Yes				Yes		
**Kim et. al.[49] (2019), South Korea, Korean**		Yes	Yes	Yes	Yes	Yes	
**Wijaya and Widiantoro[50] (2018), Taiwan, Indonesian**				Yes		Yes	Yes
**No. of studies with feature**	5	12	11	14	9	10	10

Table S3 (continue)

Author (Year), Country, Ethnicity	Tailored feedback by human coach	Progress reports/ charts	Communic-ation with peers and family	Communic-ation with health team	Presence of social comparison	Gamifi-cation	Prompts & reminders for healthy lifestyle behaviours	Total No. of features
**Bender et. al.[30] (2018), United States, Filipino American**								4
**Dong et. al.[31] (2018), China, Chinese**			Yes	Yes				5
**Dorje et. al.[32] (2019), China, Chinese**	Yes		Yes	Yes			Yes	12
**Kaur et. al.[33] (2020), India, Indian**				Yes				3
**Kim et. al.[34] (2019), South Korea, Korean**			Yes	Yes				13
**Lee et. al.[35] (2018), South Korea, Koreans**	Yes	Yes		Yes				8
**Lee et. al.[36] (2019), South Korea, Koreans**					Yes			8
**Lim et. al.[37] (2020), Singapore, Multi-racial**	Yes	Yes	Yes	Yes			Yes	15
**Muralidharan et. al.[38] (2019), India, Indian**		Yes		Yes	Yes		Yes	14
**Oh et. al.[39] (2015), South Korea, Koreans**	Yes	Yes		Yes				12
**Shin et. al.[40] (2017), South Korea, Korean**						Yes	Yes	6
**Suen et. al.[41] (2019), Hong Kong, Chinese**				Yes			Yes	7
**Tanaka et. al.[42] (2018), Japan, Japanese**	Yes		Yes	Yes	Yes			8
**Yang et. al.[43] (2017), Taiwan, Chinese**	Yes	Yes		Yes			Yes	8
**Yang et. al.[44] (2020), South Korea, Korean**	Yes			Yes				7
**Zhang et. al.[45] (2019), China, Chinese**	Yes	Yes (for BGL)	Yes	Yes				11
**Zhou et. al.[46] (2016), China, Chinese**	Yes	Yes		Yes				9
**He et. al.[47] (2017), China, Chinese**			Yes	Yes	Yes	Yes		10
**Kim et. al.[48] (2014), South Korea, Korean**	Yes			Yes				5
**Kim et. al.[49] (2019), South Korea, Korean**	Yes	Yes	Yes	Yes		Yes		12
**Wijaya and Widiantoro[50] (2018), Taiwan, Indonesian**			Yes		Yes	Yes		7
**No. of studies with feature**	11	8	9	17	5	4	6	
