# Supplementary material for: Efficacy of Interventions That Incorporate Mobile Apps in Facilitating Weight Loss and Health Behavior Change in the Asian Population: Systematic Review and Meta-analysis
Source: J Med Internet Res. 2021 Nov 16;23(11):e28185. doi: 10.2196/28185 (PMC8663646; doi:10.2196/28185)
Supplement: Multimedia Appendix 6 [file jmir_v23i11e28185_app6.pdf]

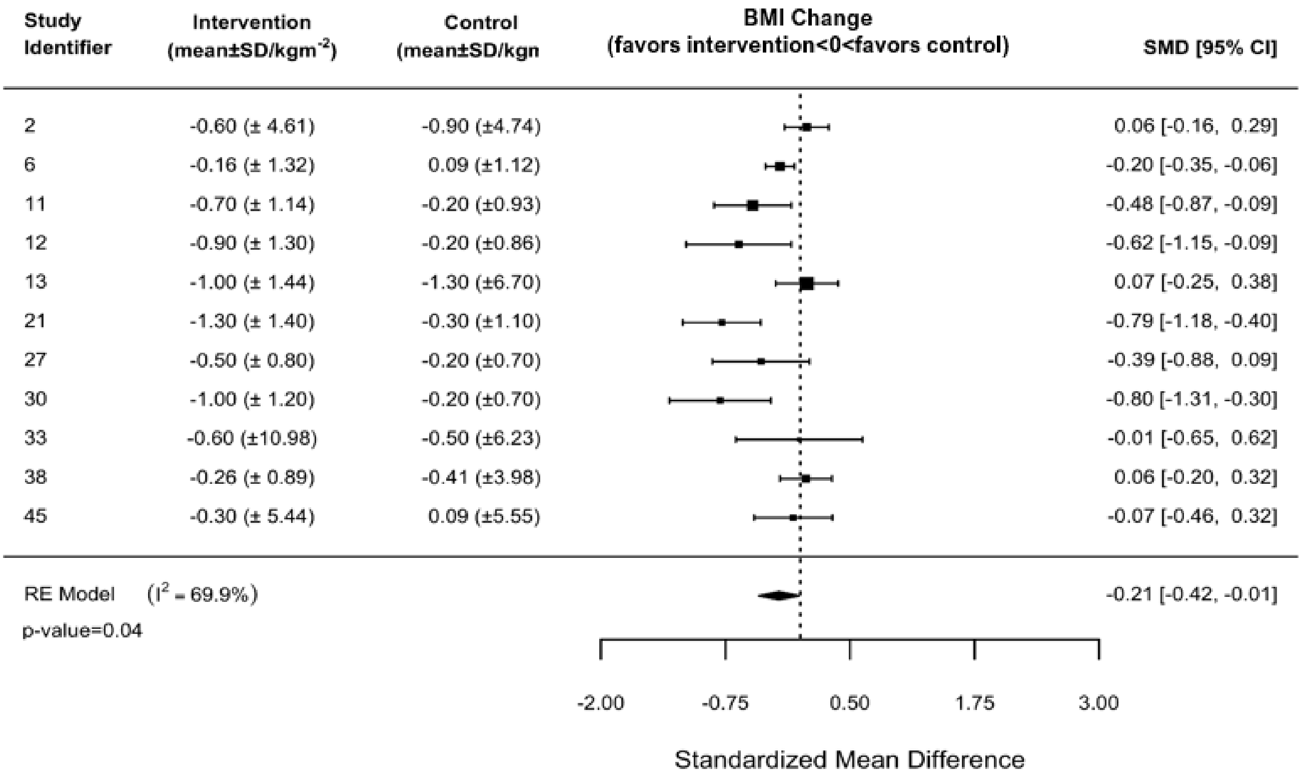

Figure S1: Forest plot showing the pooled effects of interventions incorporating app on BMI change.

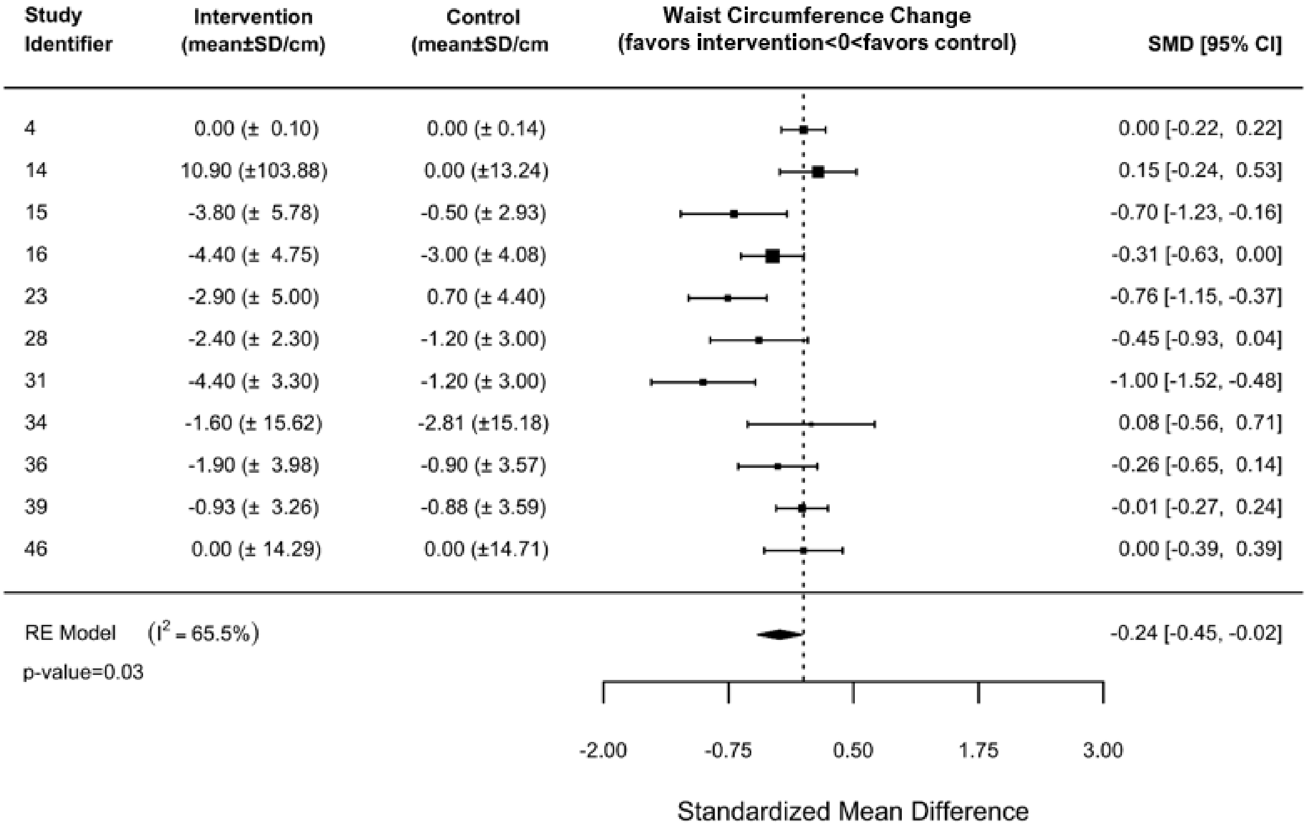

Figure S2: Forest plot showing the pooled effects of interventions incorporating app on waist circumference change.

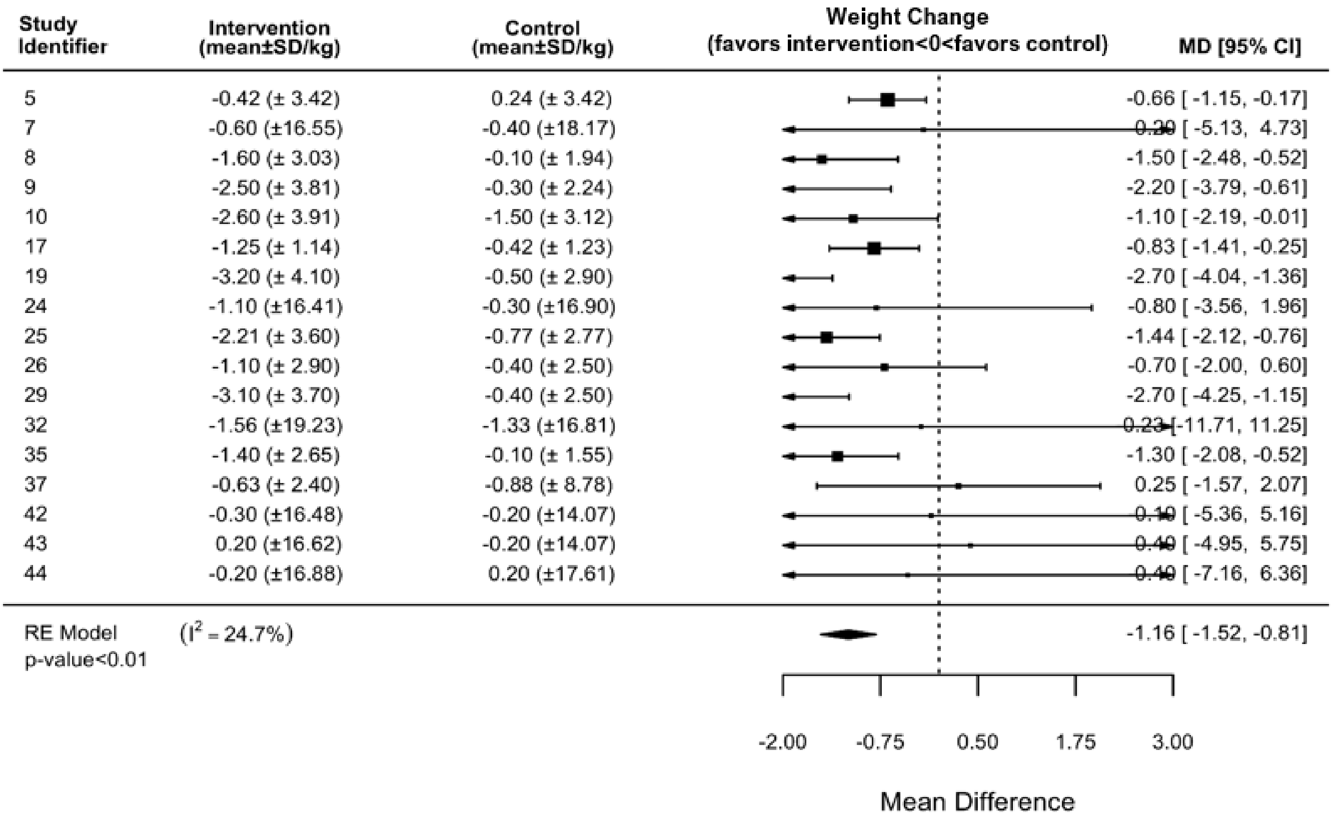

Figure S3: Forest plot showing non-standardised effects of interventions incorporating app on weight change (raw data).

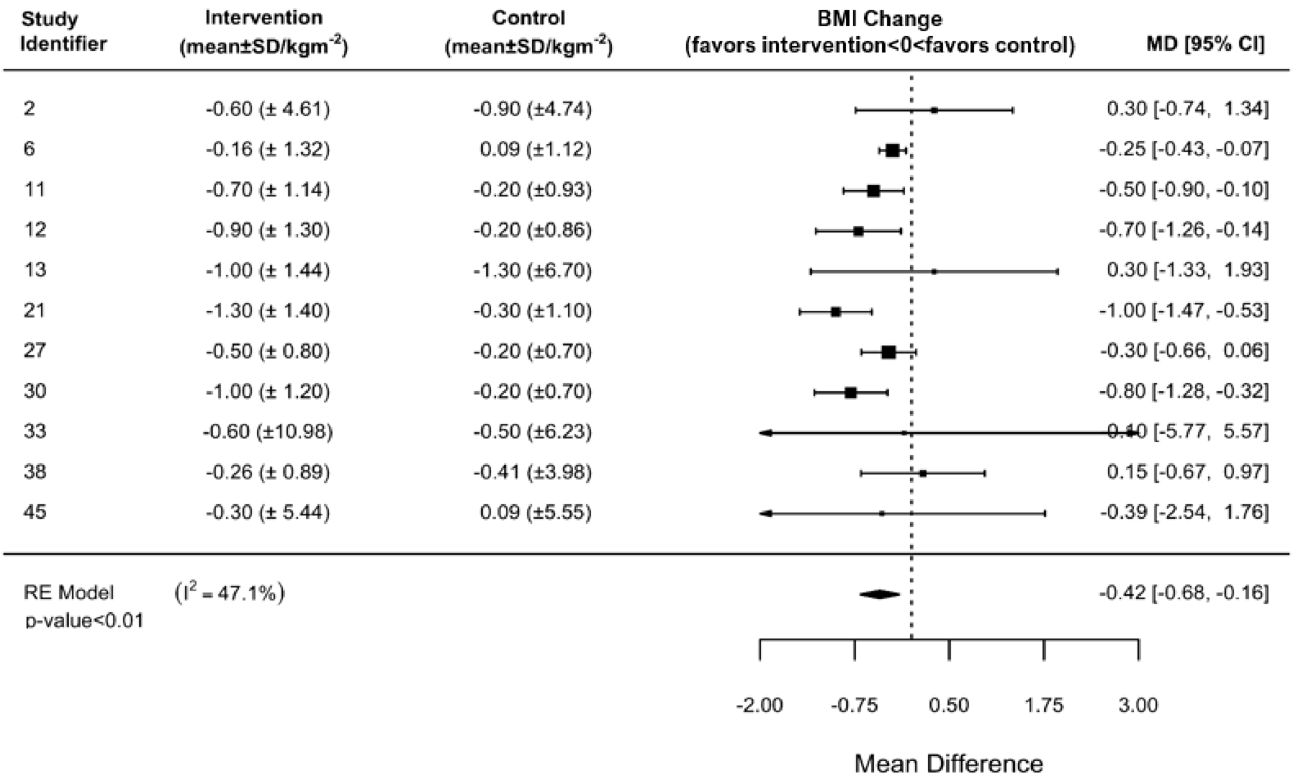

Figure S4: Forest plot showing non-standardised effects of interventions incorporating app on BMI change (raw data).

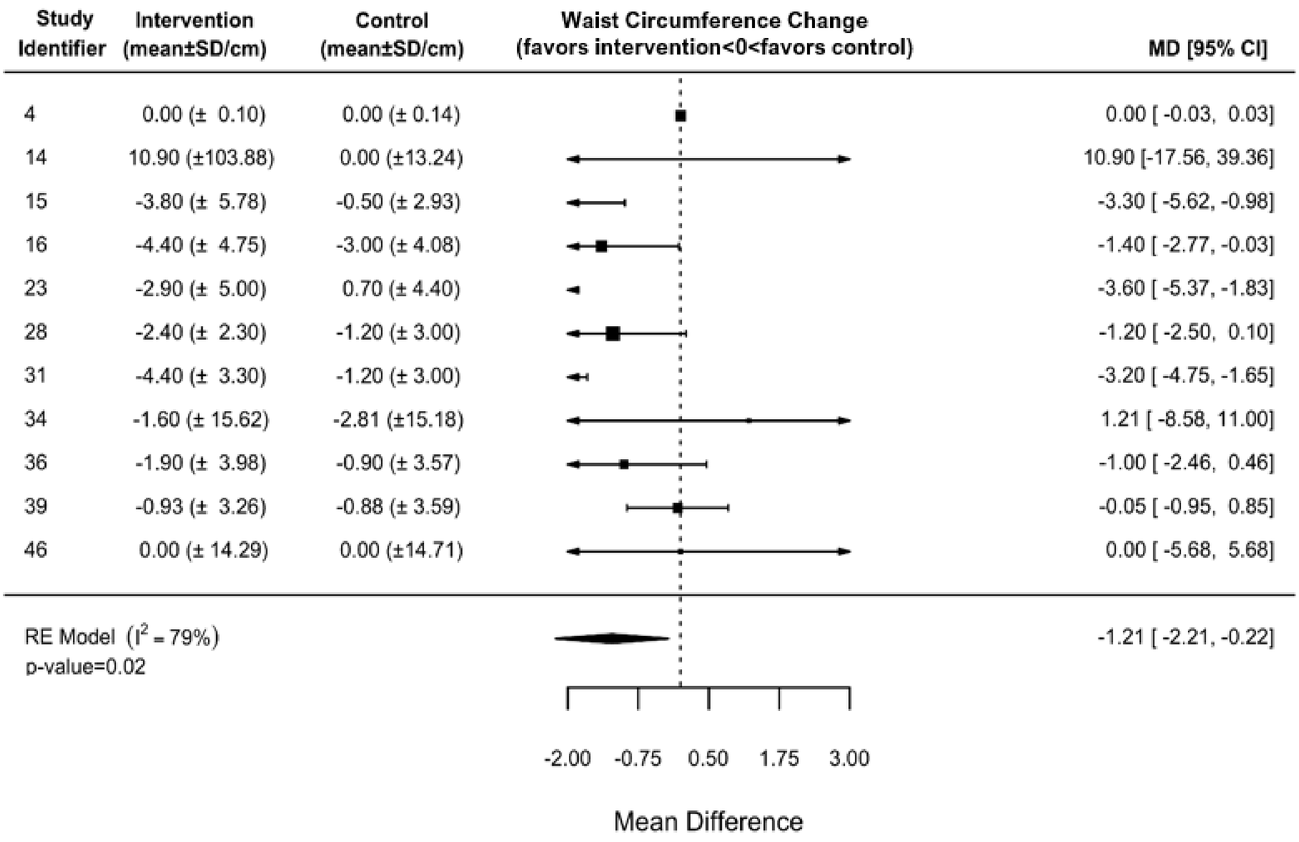

Figure S5: Forest plot showing non-standardised effects of interventions incorporating app on waist circumference change (raw data).

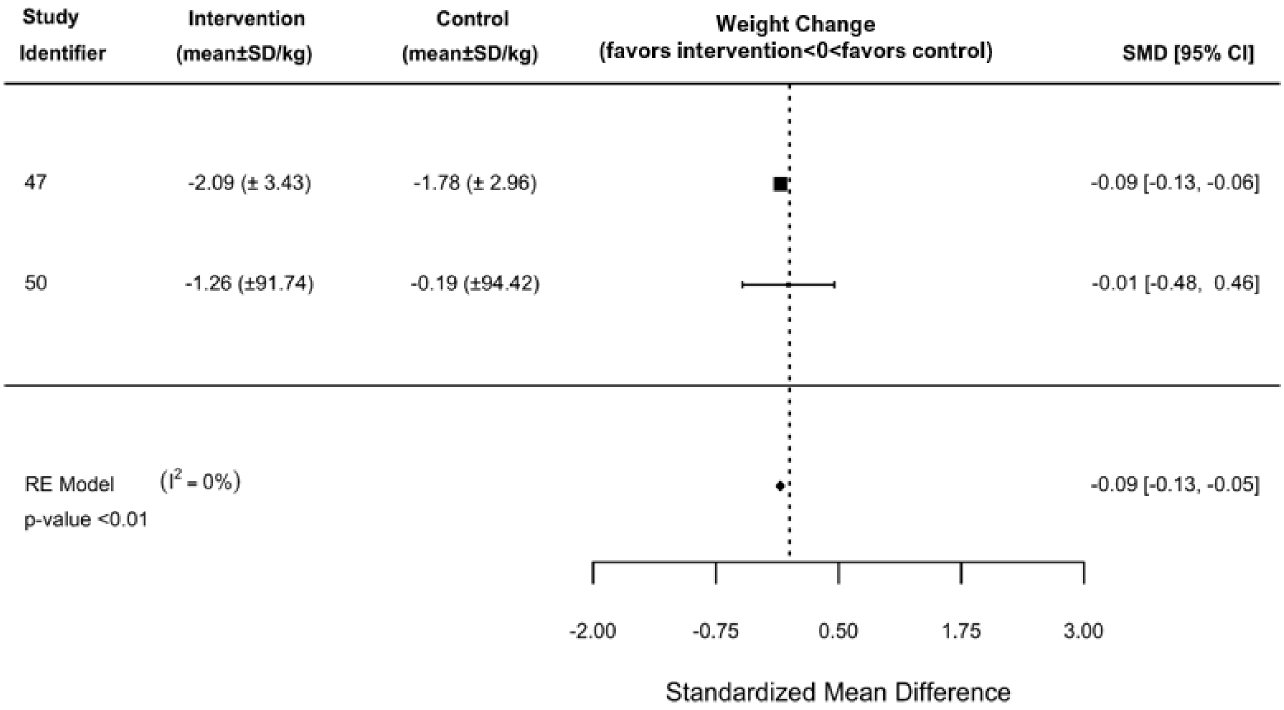

Figure S6: Forest plot showing effects of interventions incorporating app on weight change for non-randomized controlled trials.

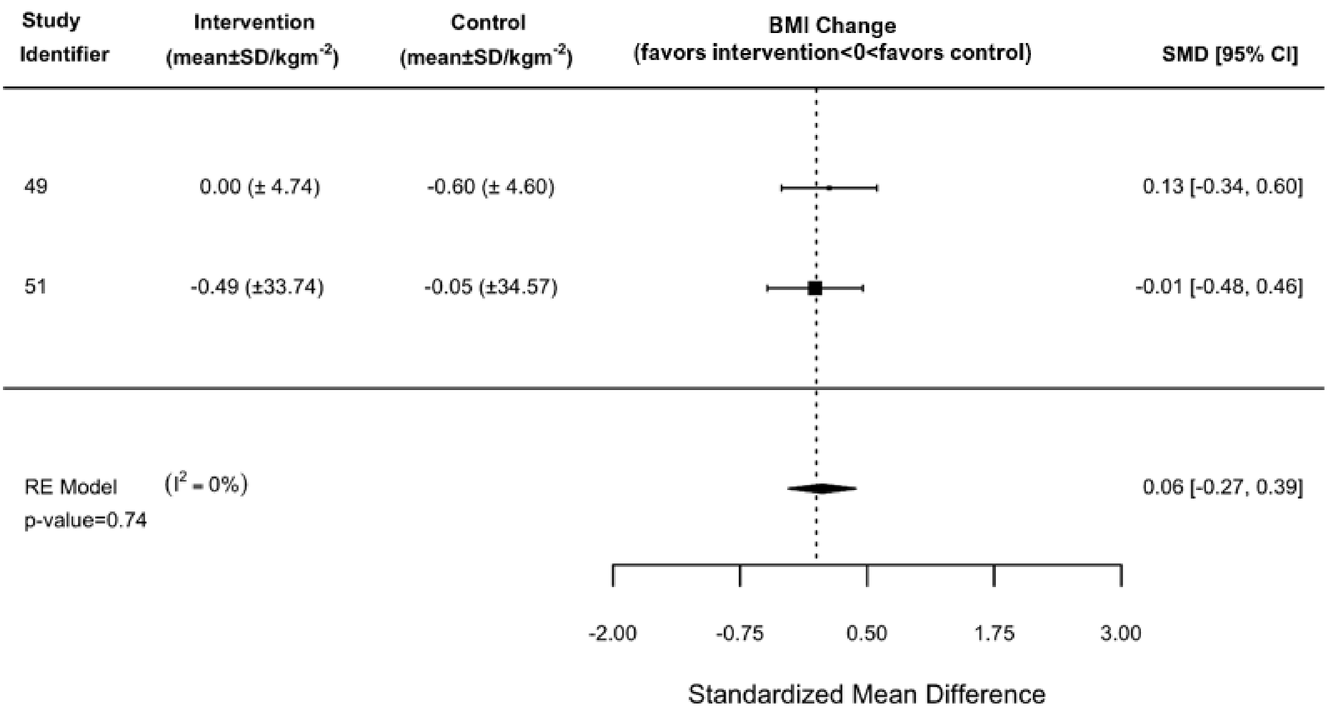

Figure S7: Forest plot showing effects of interventions incorporating app on BMI change for non-randomized controlled trials.

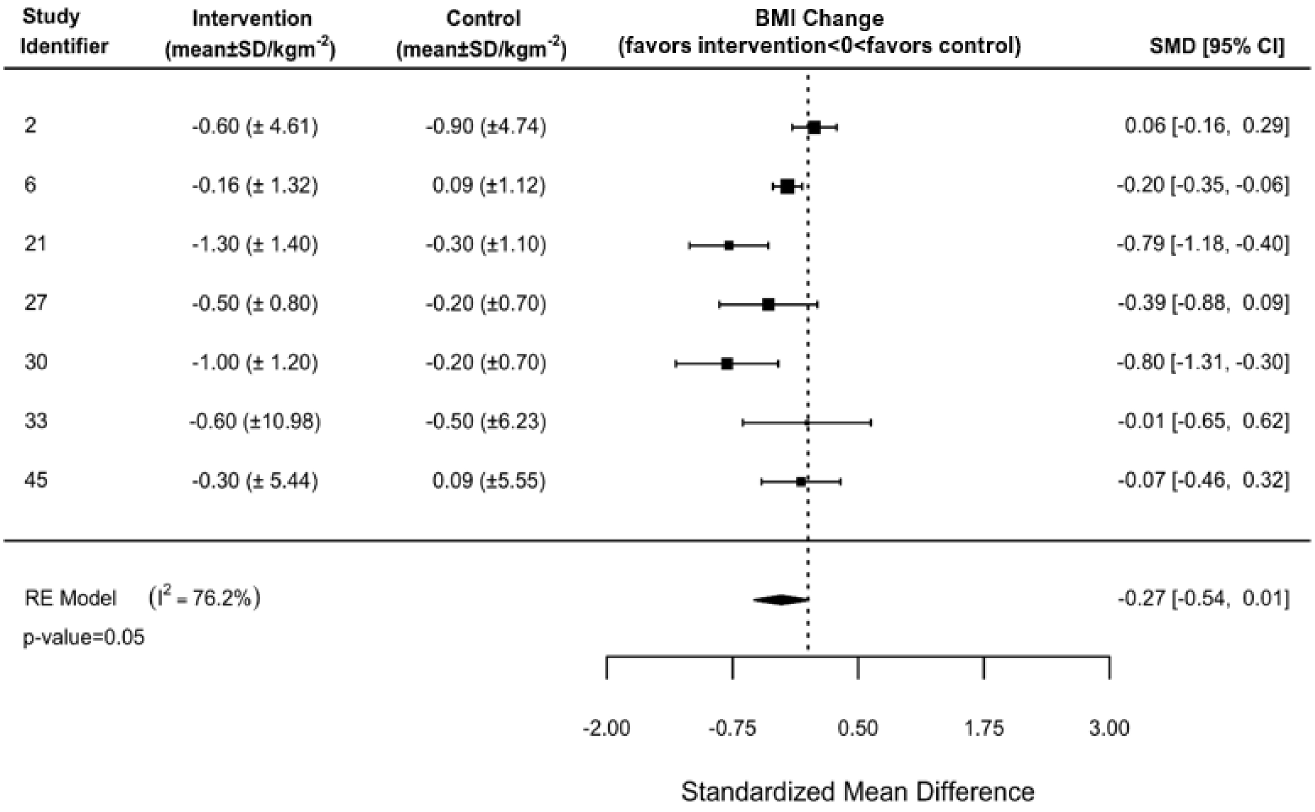

Figure S8: Forest plot showing effects of usual care with (intervention) and without (control) app on BMI change.

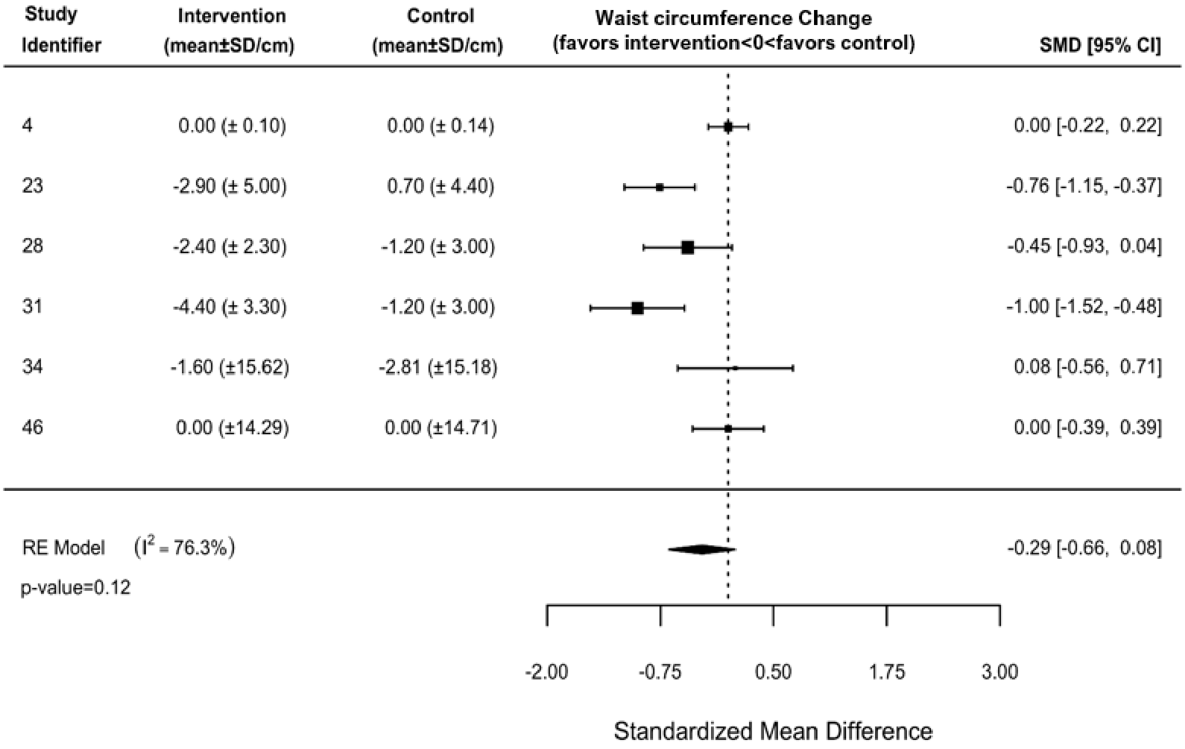

Figure S9: Forest plot showing effects of usual care with (intervention) and without (control) app on waist circumference change.

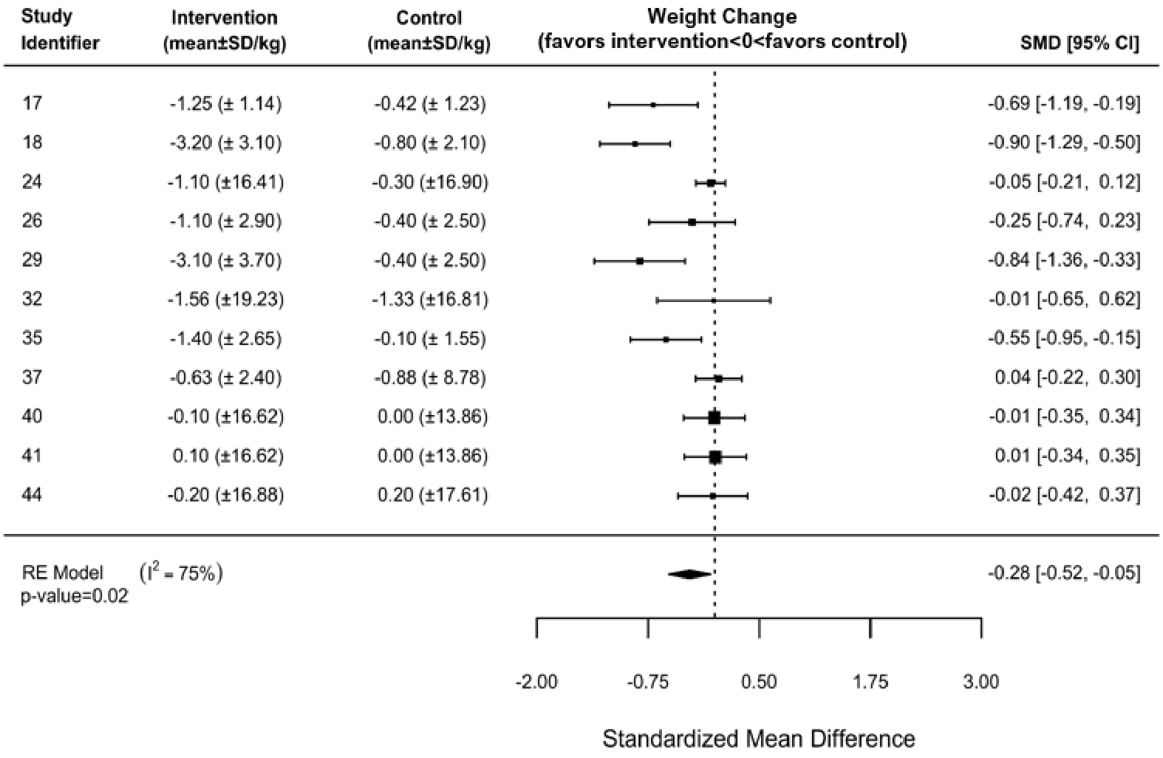

Figure S10: Forest plot showing the pooled effects of interventions incorporating app on weight change for studies with study duration of three months or less.

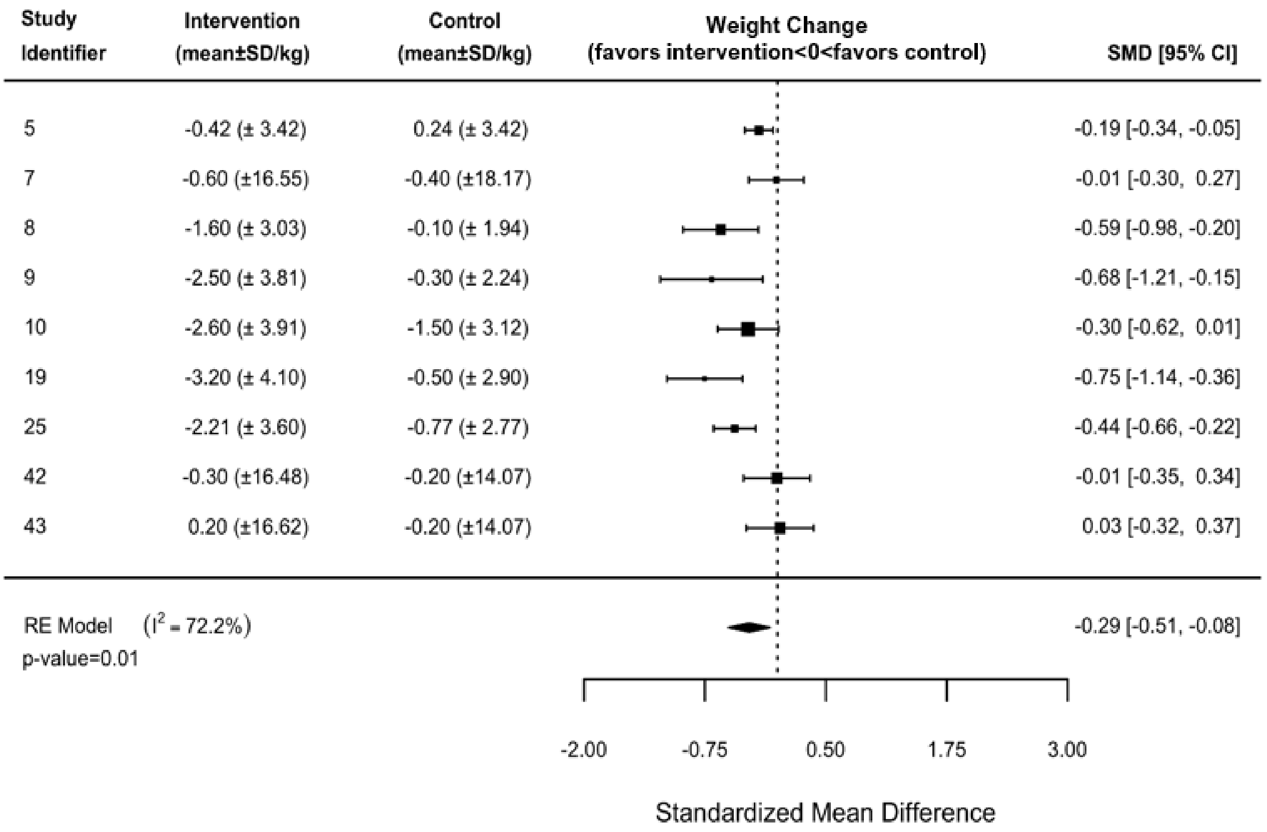

Figure S11: Forest plot showing the pooled effects of interventions incorporating app on weight change for studies with study duration between three and six months.

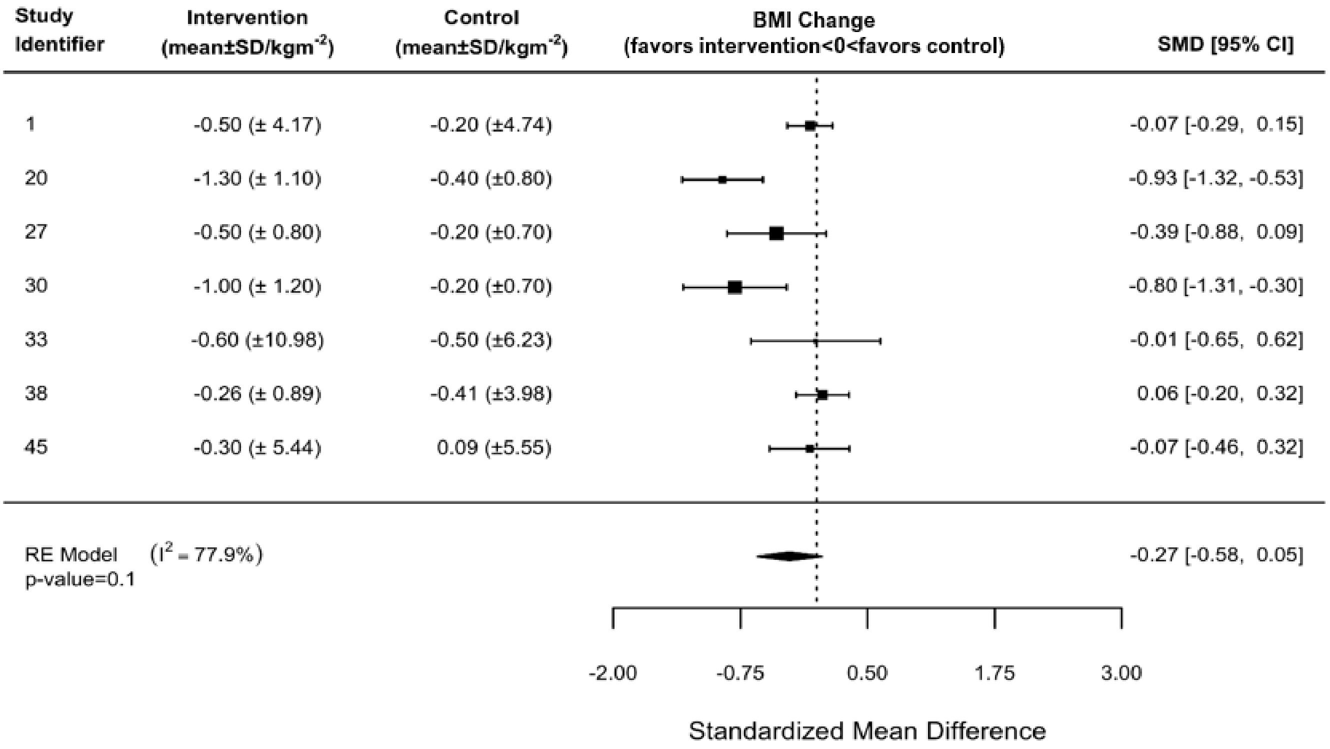

Figure S12: Forest plot showing the pooled effects of interventions incorporating app on BMI change for studies with study duration of three months or less.

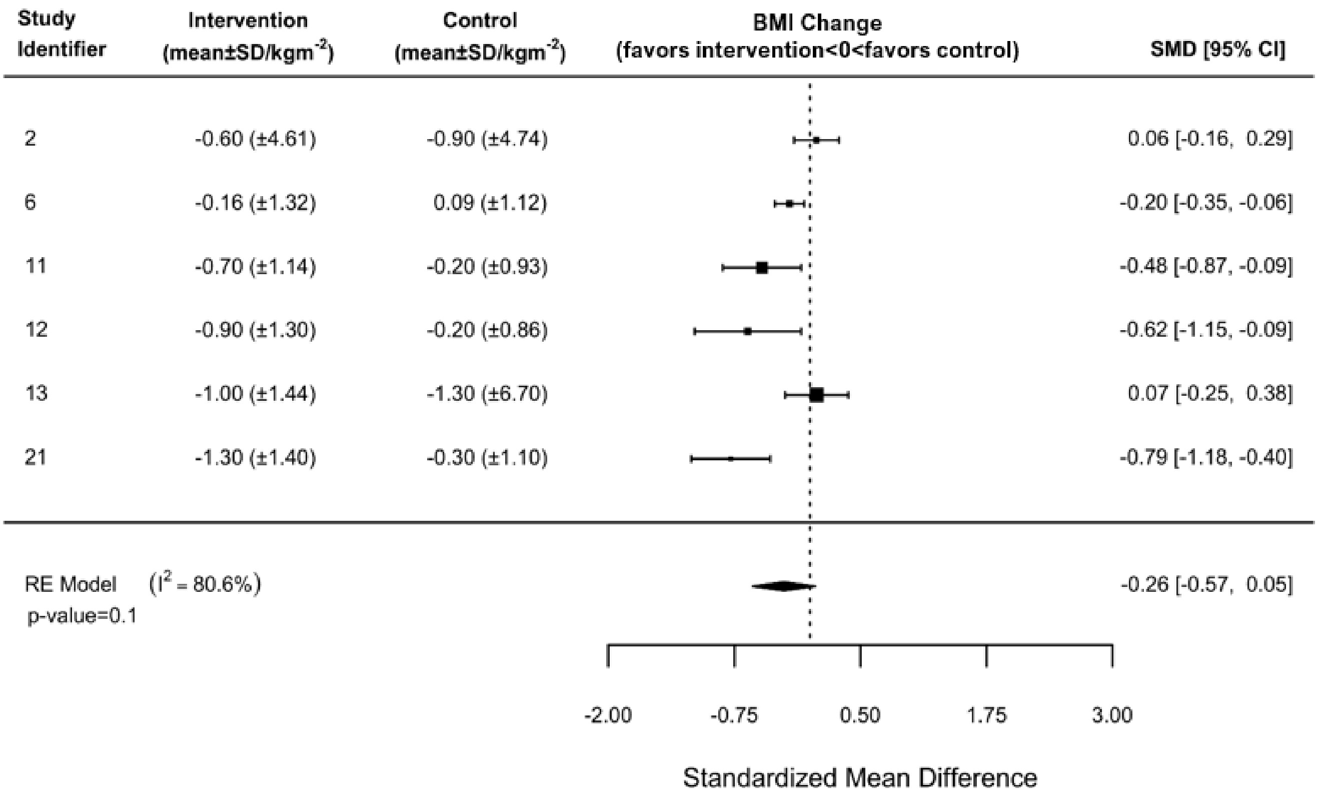

Figure S13: Forest plot showing the pooled effects of interventions incorporating app on BMI change for studies with study duration between three and six months.

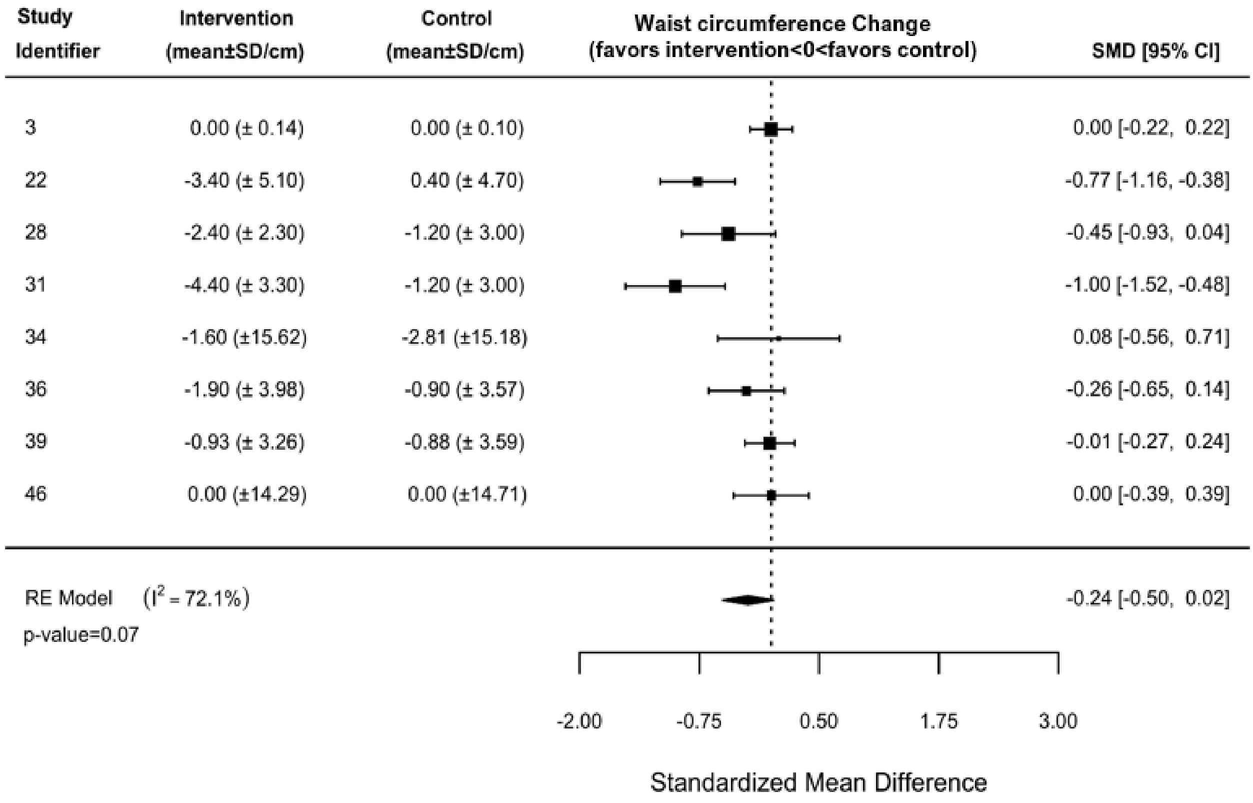

Figure S14: Forest plot showing the pooled effects of interventions incorporating app on waist circumference change for studies with study duration of three months or less.

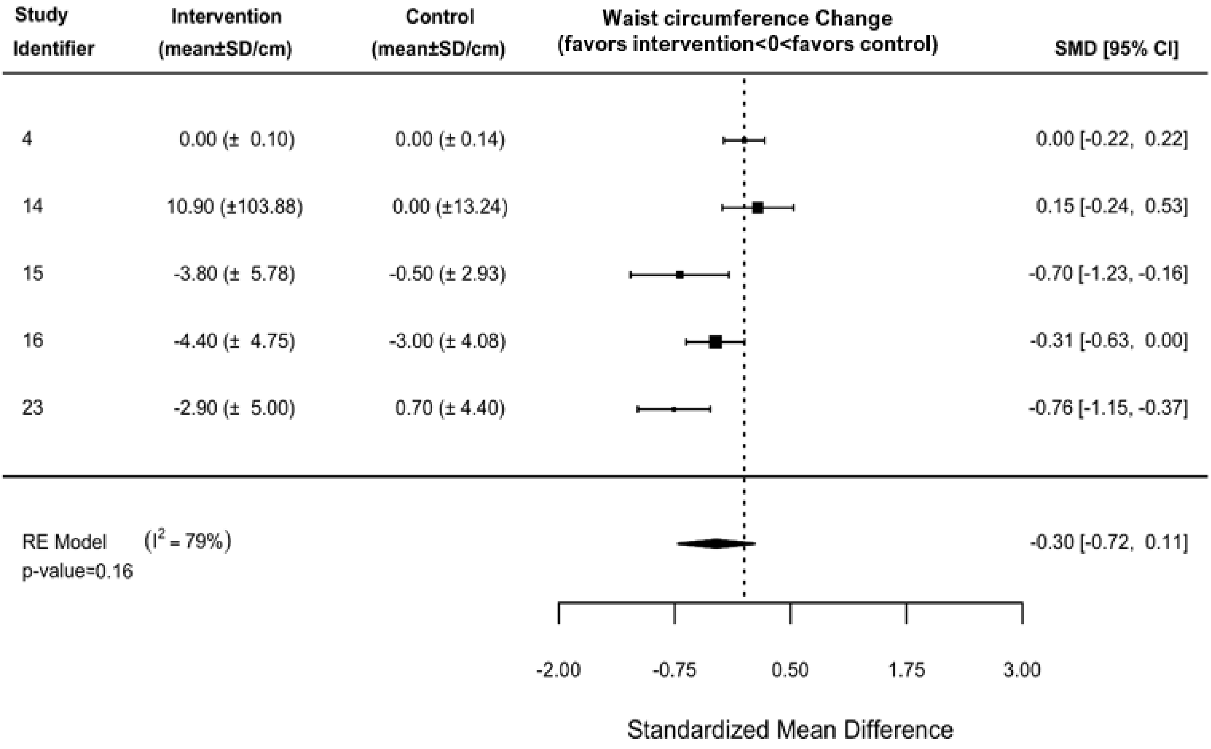

Figure S15: Forest plot showing the pooled effects of interventions incorporating app on waist circumference change for studies with study duration between three and six months.
