# Supplementary material for: Efficacy of Interventions That Incorporate Mobile Apps in Facilitating Weight Loss and Health Behavior Change in the Asian Population: Systematic Review and Meta-analysis
Source: J Med Internet Res. 2021 Nov 16;23(11):e28185. doi: 10.2196/28185 (PMC8663646; doi:10.2196/28185)
Supplement: Multimedia Appendix 7 [file jmir_v23i11e28185_app7.pdf]

Figure S3: Funnel plots of meta-analysis.

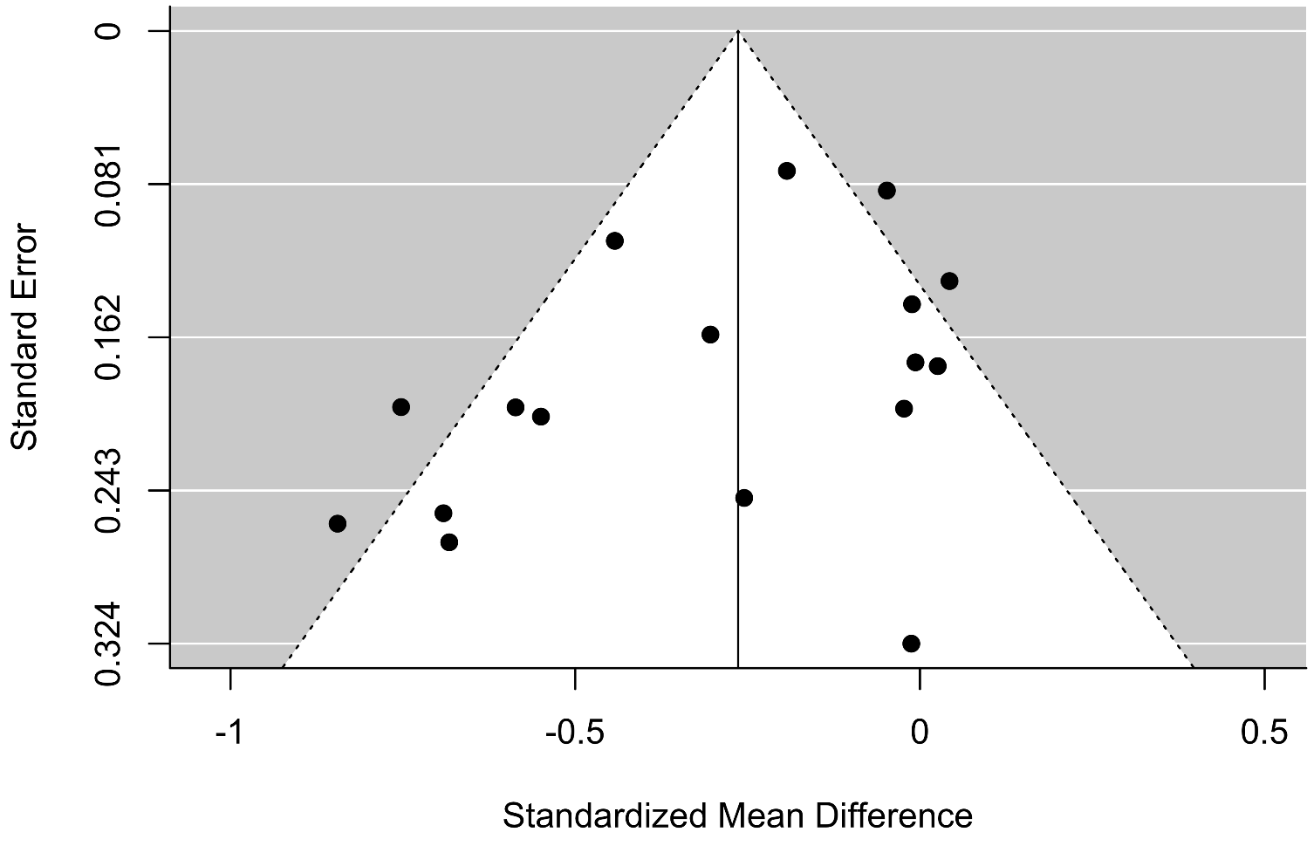

(i) Funnel plot of studies included in meta-analysis of interventions incorporating app assessing weight change.

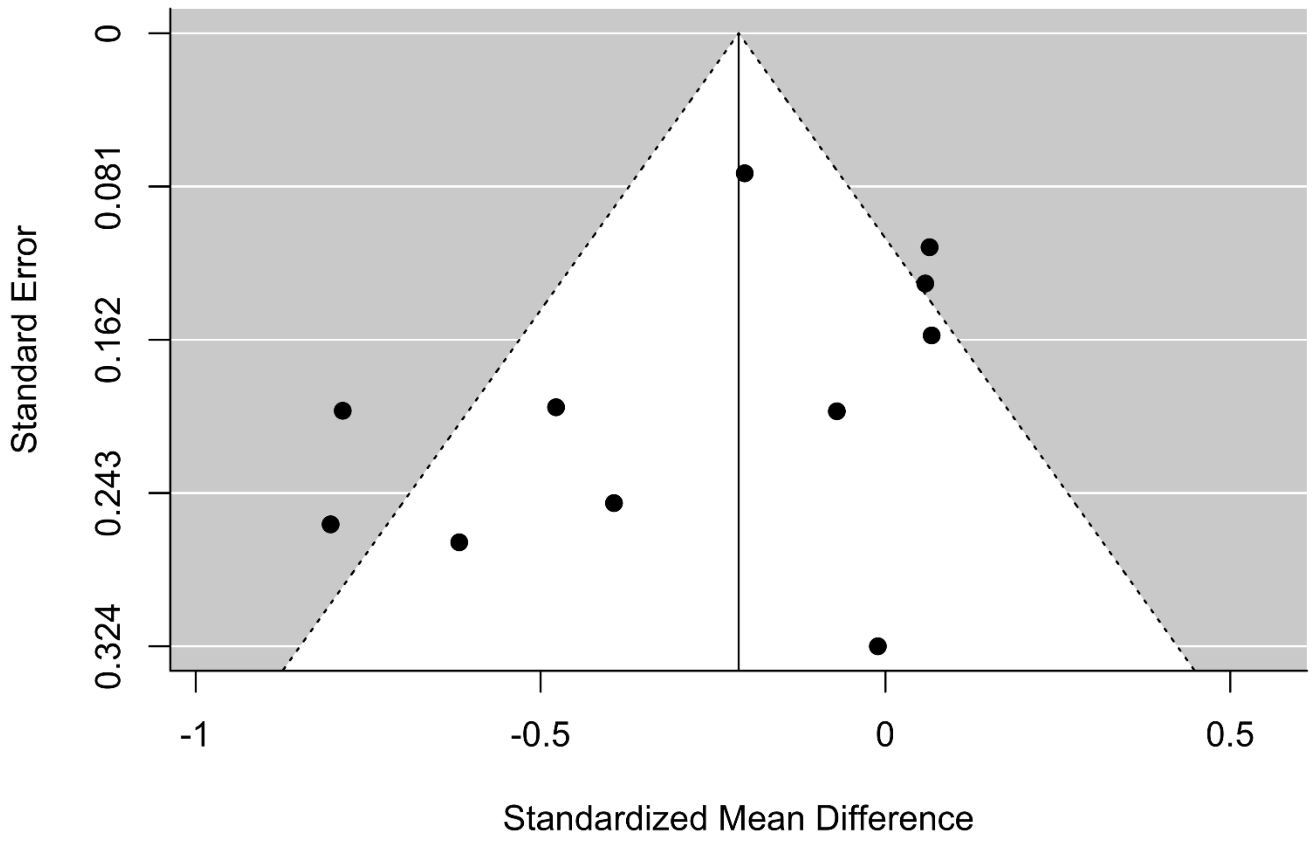

(ii) Funnel plot of studies included in meta-analysis of interventions incorporating app assessing BMI change.

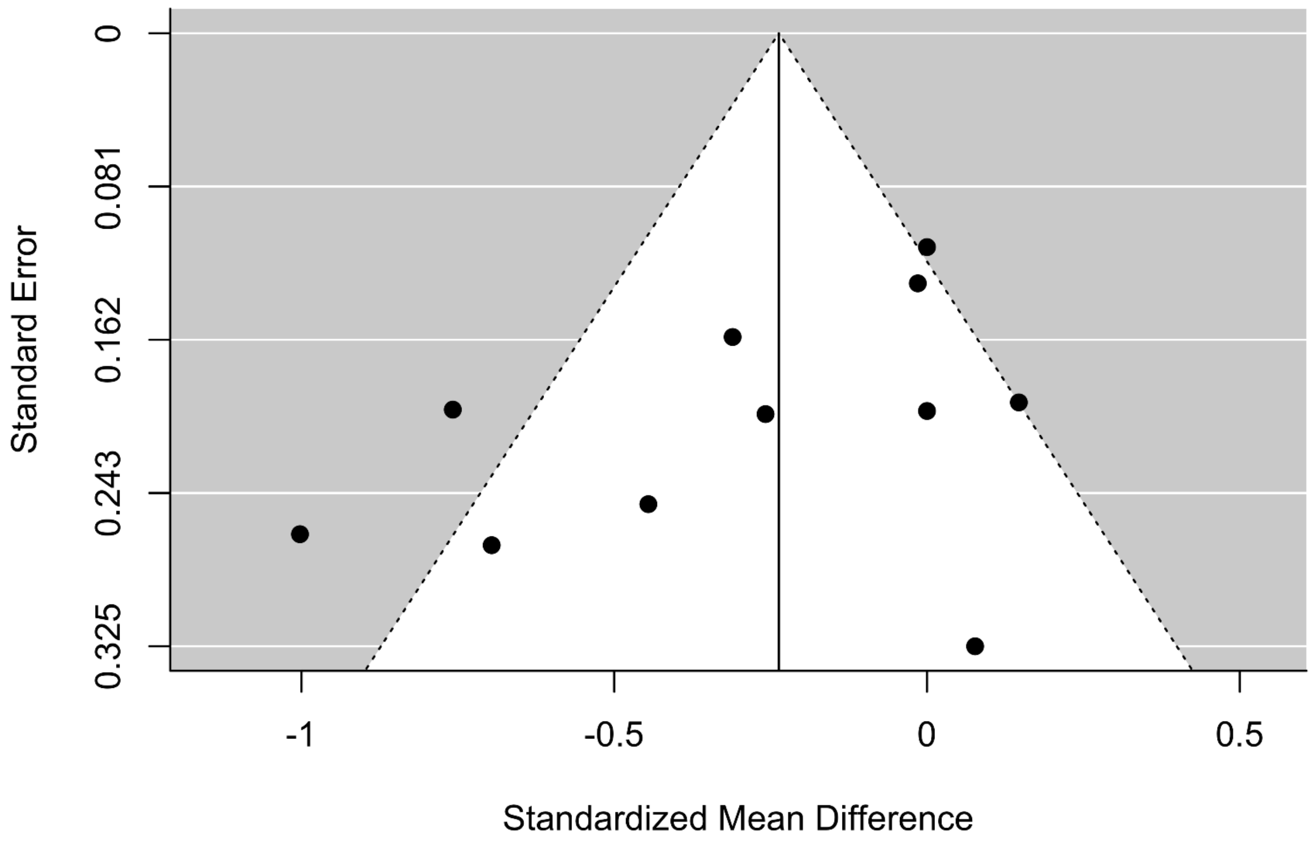

(iii) Funnel plot of studies included in meta-analysis of interventions incorporating app assessing waist circumference change.
