# Supplementary material for: Efficacy of Interventions That Incorporate Mobile Apps in Facilitating Weight Loss and Health Behavior Change in the Asian Population: Systematic Review and Meta-analysis
Source: J Med Internet Res. 2021 Nov 16;23(11):e28185. doi: 10.2196/28185 (PMC8663646; doi:10.2196/28185)
Supplement: Multimedia Appendix 8 [file jmir_v23i11e28185_app8.pdf]

Table S4: Summary of secondary outcomes including app usage and changes to lifestyle behaviours (RCTs).

Author (Year), Country, Ethnicity	Sample size, Disease group	Diet-related	Physical Activity- related	Self-efficacy- related	Smartphone usage/ engagement
**Bender et al.[30] (2018), United States, Filipino American**	n = 67<br>Overweight or obese Filipino aged ≥18 years at risk of T2DM[a] or Prediabetes	NA[b]	NA	NA	NA
**Dong et al.[31] (2018), China, Chinese**	n = 120 (119)<br>T2DM aged 18-60 years old	Diet score (mean ± SD)<br>Baseline<br>(I[c]): 5.00 ± 1.62<br>(C[d]): 4.43 ± 1.58<br>6months<br>(I): 6.12 ± 1.27<br>(C): 4.87 ± 1.43<br>1 year<br>(I): 5.75 ± 1.11<br>(C): 5.51 ± 1.32<br>Significant difference between groups (*P*<.05)<br>Significant increase in score as the follow-up increase (*P*<.05)	Exercise score (mean ± SD)<br>Baseline (I): 3.91 ± 2.72 (C): 4.29 ± 3.06<br>6 months (I): 5.68 ± 2.47 (C): 5.10 ± 2.85<br>1 year (I): 6.20 ± 2.35 (C): 5.38 ± 2.59<br><br>No significant difference between groups<br>Significant increased with time (*P*<.05)	Self-Efficacy score (mean ± SD)<br>Baseline<br>(I): 23.61 ± 5.93<br>(C): 22.74 ± 6.07<br>6 months<br>(I): 29.59 ± 4.88<br>(C): 25.10 ± 6.56<br>1 year<br>(I): 30.56 ± 4.43<br>(C): 28.21 ± 5.05<br><br>Significant difference between groups (*P*<.05)<br>Significant increase in score as the follow-up increase (*P*<.05)	NA
**Dorje et al.[32] (2019), China, Chinese**	n = 312<br>Coronary heart disease patients aged ≥18 years	Dietary habits (World Health Organization Steps Instrument)<br>122 out of 125 intervention participants reported they are eating more healthily after receiving intervention.	Increased walking distance in 6-minute walk test (mean ± SD)<br>Baseline (I): 489.2 ± 99.4 (C): 485.0 ± 93.5<br>2 months (I): 539.1 ± 68.0 (C): 517.8 ± 74.6<br>2 months adjusted mean: 20.64 ± 7.50 to 33.77 (*P*=.03)<br>6 months (I): 543.4 ± 67.5 (C): 523.5 ± 60.2<br>6 months adjusted mean: 22.29 ± 8.19 to 36.38 (*P*=.03)<br><br>120 out of 125 intervention participants reported they had increased physical activity frequency and intensity after receiving intervention.	NA	1. 119 (95%) of 125 intervention participants indicated that they had read more than 75% of the modules and messages, 87 (70%) had shared the modules and messages with family members or friends<br>2. 123 (98%) of out 125 wanted to use app beyond 6 months

Table S4 (continue)

Author (Year), Country, Ethnicity	Sample size, Disease group	Diet-related	Physical Activity- related	Self-efficacy- related	Smartphone usage/ engagement
**Kaur et al.[33] (2020), India, Indian**	n = 732<br>Adults aged 35-70 years	Actual dietary intake changes (mean, 95% CI)<br>**Calorie intake**<br>I: -332.48 (-383.3, -281.6) *P*<.001<br>C: -0.96 (-29.6, 27.7) *P*=.95<br>Net: -331.52 (-389.6, -273.4) *P*<.001<br>**Fat intake**<br>I: -14.27 (-16.4, -12.2) *P*<.001<br>C: -1.75 (-3.1, -0.4) *P*=.01<br>Net: -12.52 (-15.0, -10.1) *P*<.001<br>**Sugar intake**<br>I: -13.43 (-15.4, -11.5) *P*<.001<br>C: -2.01 (-3.1, -0.9) *P*<.001<br>Net: -11.42 (-13.6, -9.2) *P*<.001<br>**Salt intake**<br>I: -1.01 (-1.2, -0.8) *P*<.001<br>C: -0.50 (-0.7, -0.3) *P*<.001<br>Net: 0.51 (-0.8, -0.2) *P*<.001<br>**Fruit & Vegetables intake**<br>I: +38.96 (27.4, 50.5) *P*<.001<br>C: -32.61 (-43.3, -21.9) *P*<.001<br>Net: +71.57 (55.8, 87.3) *P*<.001	NA	Significant increase in ASE (attitude, social-influence and self-efficacy) score in the intervention group compared to the comparison group	NA
**Kim et al.[34] (2019), South Korea, Korean**	n = 191 (172)<br>Stable T2DM aged 19-80 years old with HbA_{1c}[e] between 7%-10%	Diet component of SDSCA[f] score did not change significantly at week 24 in either group compared with week 0.	Physical activity component of SDSCA score did not change significantly at week 24 in either group compared with week 0.	The SDSCA scores did not change significantly at week 24 in either group compared with week 0.	NA
**Lee et al.[35] (2018), South Korea, Koreans**	n = 422 (324)<br>Overweight or obese university medical school students with Metabolic Syndrome	NA	IPAQ[g]<br>Participants in the intervention group had higher levels of health-enhancing physical activity than the control group.<br>Nil significant differences between group.	NA	NA

Table S4 (continue)

Author (Year), Country, Ethnicity	Sample size, Disease group	Diet-related	Physical Activity- related	Self-efficacy- related	Smartphone usage/ engagement
**Lee et al.[36] (2019), South Korea, Koreans**	n = 65<br>Individuals aged 20-65 years with colorectal polyps diagnosis within the last 2 years of the study	Actual dietary intake changes via validated Food Frequency Questionnaire (mean ± SD)<br>**Fat intake**<br>I: −0.44 ± 0.72 (*P*<.01)<br>C: −0.29 ± 1.01 (*P*=.13)<br>Between group *P*=.28<br>Odds Ratio 0.81<br><br>**Vegetables intake**<br>I: 0.81 ± 1.00 (*P*<.01)<br>C: 0.52 ± 1.12 (*P*=.02)<br>Between group *P*=.14<br>Odds Ratio 0.59<br><br>Actual dietary intake changes via validated Food Frequency Questionnaire (mean ± SD)<br>**Fruit intake**<br>I: 0.31 ± 0.78 (*P*=.03)<br>C: 0.13 ± 1.02 (*P*=.62)<br>Between group *P*=.27<br>Odds Ratio 0.81<br><br>Nil significant difference between groups for all.	Godin Leisure-Time Exercise Questionnaire (mean ± SD)<br>Change in number of strenuous-intensity exercises > 15minutes/day per week<br>I: 0.94 ± 1.13 (*P*<.01)<br>C: 0.58 ± 1.21 (*P*=.01)<br><br>Nil significant difference between groups, *P*=.20	NA	1. Adherence was defined as using a device or diary for more than two thirds of the follow-up period, which was assessed daily (nil further specified)<br>2. Use of the smartphone app was not associated with changes in physical activity, lower fat intakes, or higher vegetable and fruit intakes from baseline.<br>3. When analysis was restricted to the group with strict adherence, the researchers found that use of the smartphone app was associated with weight reduction compared with the control group (−2.1 vs −0.6 kg: *P*<.01) In addition, the smartphone app significantly raised the level of physical activity (1.6 vs 0.7 times/week; *P*=.03) and frequency of vegetable intake (1.4 vs 0.8 times/week; *P*=.03) when limited to the strict adherence group
**Lim et al.[37] (2020), Singapore, Multi-racial**	n = 108<br>Overweight or obese Non-Alcoholic Fatty Liver Disease patients aged 21-70	NA	NA	NA	1. High percentage of active users in the intervention, with 76% (37/49) of participants showing a daily log-in of 137 days over a 182-day period (>75.3% of the total time)<br>2. Average log-in days for the first 3 months, 4-6 months, and overall were 79.6 days (SD 17.9), 71.1 days (SD 25.6), and 151 days (SD 41.1), respectively.<br>3. Mean percentage of log-in days was 87.6% (SD 19.6) in the first 3 months and decreased to 78.1% (SD 28.2) at 4-6 months.<br>4. Meal and weight logging were at 56.7% (SD 51.6) and 77.0% (SD 28.5) of the recommended utilization rate of daily and twice a week, respectively

Table S4 (continue)

Author (Year), Country, Ethnicity	Sample size, Disease group	Diet-related	Physical Activity- related	Self-efficacy- related	Smartphone usage/ engagement
**Muralidharan et al.[38] (2019), India, Indian**	n = 741 (5610<br>Overweight or obese aged 20-65 years with prediabetes	NA	NA	NA	NA
**Oh et al.[39] (2015), South Korea, Korean**	n = 422 (334)<br>Obese adults aged 20-70 with BMI ≥25 kg/m² and diagnosed with Metabolic syndrome	3-Day Diet Recall<br>Diet habit improve from baseline to week 24 were superior in the intervention group compared with the control group (diet habit *P*=.01)	IPAQ<br>Physical activity were not statistically significantly different between the groups	NA	NA
**Shin et al.[40] (2017), South Korea, Korean**	n = 105 (98)<br>Overweight or obese males aged 19-45 years with BMI ≥27 kg/m²	No significant differences in total calorie intake between groups.	IPAQ<br>Increase in physical activity per week in calorie expenditure (mean ± SD)<br>I1: 3747.9 ± 5954.8<br>I2: 304.6 ± 3129.1<br>C: 532.3 ± 2050.1<br><br>Changes in physical activity were significantly higher in group with app + financial incentives relative to that of group with app alone and control (*P*s<.01)	NA	1. Analysis of activity tracker data showed that the total number of days involving exercise goal completion was strongly correlated with final weight reduction completion.<br>2. Multilevel analysis showed that the probability of daily physical activity goal achievement was higher for group with app and financial incentives relative to that of just app alone, but this decreased gradually as the study progressed
**Suen et al.[41] (2019), Hong Kong, Chinese**	n = 59<br>Healthy overweight or obese adults aged ≥ 18 years with BMI ≥25 kg/m², without ear injuries	Fullness Rating<br>**(Lunch)**<br>(I): +1.06 (*P*=.047)<br>(C): +0.60 (*P*=.33)<br>No significant differences between groups<br>**(Dinner)**<br>(I): +1.40 (*P*=0.01)<br>(C): +0.71 (*P*=0.32)<br>No significant differences between groups	NA	NA	NA

Table S4 (continue)

**Author (Year), Country, Ethnicity**	**Sample size, Disease group**	**Diet-related**	**Physical Activity- related**	**Self-efficacy- related**	**Smartphone usage/ engagement**
**Tanaka et al.[42] (2018), Japan, Japanese**	n = 112<br>Overweight, obese, or abdominally obese adults aged 20-64 years with cardiometabolic risk factor(s) or metabolic syndrome	Obesogenic eating behaviour (Percentage improved in I vs C):<br>-Eating snacks ≥ once a week (16.9% vs 6.1%)<br>-Eating out ≥ once a week (4.2% vs 3.0%)<br>-Eating fast foods ≥ once a week (19.7% vs 6.1%)<br>-Eating confectioneries ≥ once a week (8.5% vs 3.0%)<br>-Drinking Sugar Sweetened Beverages ≥ once a week (14.1% vs 3.0%)<br>-Eating until full (14.1% vs 6.1%)<br>-Binge eating (23.9% vs 12.1%)<br><br>Nil significant changes between group	NA	NA	1. Recorded the total number of meal photo uploads as a proxy measure of study adherence.<br>2. Median (first–third quartiles) frequency of meal photo uploads per participant was 82 times (23–139.5 times) during the 8-week period. (total possible 168 times)<br>3. One-fourth or more of the participants did not upload any meal photos in each 10-day interval<br>4. Frequencies of photo uploads every 10 days per participant were 17 times (5.5–25.5 times) for the initial 10 days, 16 times (4–28 times) for days 11 to 20, 17 times (1–25 times) for days 21 to 30, 17 times (0–25 times) for days 31 to 40, 15 times (0–25.5 times) for days 41 to 50, and 3 times (0–18.5 times) for day 51 to the end of the program.<br>5. As compared to the first tertile (<29 times) of the photo upload frequency, weight loss for the second (29–127 times) and the third (≥128 times) tertiles exhibited 1.9 kg (95% CI: 0.6, 3.2 kg) and 3.5 kg (95% CI: 2.2, 4.8 kg), respectively.
**Yang et al.[43] (2017), Taiwan, Chinese**	n = 53 (46)<br>Overweight or obese people with BMI ≥24kg/m^2 and metabolic abnormalities	NA	Total physical activity hours per week via physical activity sensor (mean ± SD)<br>Baseline (I): 35.83 ± 1.58<br>(C): 36.84 ± 2.00<br>6 months (I): 37.25 ± 1.65<br>(C): 35.96 ± 1.82<br>Significant differences between group (*P*<.001)<br><br>Sedentary hours per week via physical activity sensor (mean ± SD)<br>Baseline (I): 9.51 ± 1.22 (C): 8.55 ± 1.15<br>6 months (I): 7.88 ± 1.44 (C): 9.22 ± 1.28<br>Significant differences between group (*P*<.001)	NA	NA

Table S4 (continue)

**Author (Year), Country, Ethnicity**	**Sample size, Disease group**	**Diet-related**	**Physical Activity- related**	**Self-efficacy- related**	**Smartphone usage/ engagement**
**Yang et al.[44] (2020), South Korea, Korean**	n = 247 (239)<br>Adults aged ≥18 years with T2DM for ≥1 year and HbA_{1c} 7%-10%	NA	NA	NA	NA
**Zhang et al.[45] (2019), China, Chinese**	n = 234 (194)<br>Adults aged 18-65 years with diagnosed diabetes for more than 6 months and has HbA_{1c} ≥8%	NA	NA	NA	1. Frequencies of app usage in group with a physician and group with interactive health team were 10.7 (SD 9.5) and 11.1 (SD 7.3) times per week. The difference was not significant (P = .83).<br>2. In group with interactive health team, each patient received an average of 30.5 (SD 3.6) times of interactive management during the whole follow-up, with an average guiding time of 458 (SD 54) minutes.
**Zhou et al.[46] (2016), China, Chinese**	n = 100<br>Adults aged 18-74 years with diagnosed diabetes without severe complications	Overall SDSCA score which includes dietary habits (mean ± SD)<br>Baseline<br>(I): 12.5 ± 6.4<br>(C): 12.8 ± 7.0<br>3 months<br>(I): 28.3 ± 4.4<br>(C): 23.0 ± 6.3<br>Significant different within intervention and control group (*P*<.01)<br>Significant difference between group (*P*<.01)	Overall SDSCA score which includes physical activity (mean ± SD)<br>Baseline<br>(I): 12.5 ± 6.4<br>(C): 12.8 ± 7.0<br>3 months<br>(I): 28.3 ± 4.4<br>(C): 23.0 ± 6.3<br>Significant different within intervention and control group (*P*<.01)<br>Significant difference between group (*P*<.01)	SDSCA Self-care behaviour scores (mean ± SD)<br>Baseline<br>(I): 12.5 ± 6.4<br>(C): 12.8 ± 7.0<br>3 months<br>(I): 28.3 ± 4.4<br>(C): 23.0 ± 6.3<br>Significant different within intervention and control group (*P*<.01)<br>Significant difference between group (*P*<.01)	1. Mean number of contacts with intervention patients over the 3-month period was 11 ± 3 times, while the minimum was 7 times, and the maximum was 18.

Table S4 (continue)

Author (Year), Country, Ethnicity	Sample size, Disease group	Diet-related	Physical Activity- related	Self-efficacy- related	Smartphone usage/ engagement
**He et al.[47] (2017), China, Chinese**	n = 15,818 (15,310)<br>General population aged ≥18 years who are keen on weight loss	NA	NA	NA	1. Messages were read more than 247,000 times and were sent to other Wechat accounts more than 6500 times.<br>2. 3620 participants communicated with others in “microcommunity discussions” and there were more than 20,000 posts.<br>3. Experts were consulted online more than 14,576 times and 28,000 questions were asked, among which 270 representative questions were responded to in detail.<br>4. More than 8200 people regularly received feedback on their weight, diet, and exercise data and conducted self-assessments via Wechat.<br>5. Wechat activeness was represented by Wechat cumulative scores, which were related to actual official Wechat account use. Higher scores indicated that an individual was more active in using Wechat to lose weight. Scores less than or equal to 50 were regarded as inactive, and scores of 50 or more were active. A significant number of participants in the Wechat group were inactive (83.18%, 9852/11,843) and only 16.8% (1991/11,843) were active.
**Kim et al.[48] (2014), South Korean, Korean**	n = 73 (70)<br>Adults aged 20-70 years with T2DM for >1 year with HbA_{1c} 7%-10% at baseline	NA	NA	NA	NA

Table S4 (continue)

Author (Year), Country, Ethnicity	Sample size, Disease group	Diet-related	Physical Activity- related	Self-efficacy- related	Smartphone usage/ engagement
**Kim et al.[49] (2019), South Korea, Korean**	n = 1117<br>(C): Individuals with metabolic abnormalities according to the Adult Treatment Panel (ATP) III criteria.<br><br>(I): Recruited from those who had previously completed a 24-week mobile service program as part of the First-Year Public Health Centre Mobile Healthcare pilot project.	Mini dietary assessment scores (mean ± SD)<br>Baseline (I): 4.53 ± 0.10 (C): 4.77 ± 0.16<br>24-weeks (I): 5.57 ± 0.08 (C): 5.13 ± 0.14<br>Significant difference within intervention group (*P*=.001) and between group (*P*<.001)<br><br>Intervention group achieved significant difference within group for:<br>Low-sodium preference, reading nutrition label, eating breakfast, eating grains and wholegrains, 2 types of vegetables, fruits, consume dairy daily, balanced diet, limit salt or soy sauce, remove fat from meat, fewer fried food<br><br>Intervention group achieved significant difference between group for:<br>Reading nutrition label, eating 2 types of vegetables, consume dairy daily	Number of individuals who perform moderate-intensity physical activity (mean ± SD)<br>Baseline (I): 109 ± 11.96 (C): 38 ± 18.45<br>24-weeks (I): 155 ± 17.01 (C): 37 ± 17.96<br>Intervention group achieved significant difference within group (*P*<.001) but nil significant difference between group<br><br>Number of individuals who walked (mean ± SD)<br>Baseline (I): 404 ± 44.35 (C): 75 ± 36.41<br>24weeks (I): 539 ± 59.17 (C): 96 ± 46.60<br><br>Intervention and control group both achieved significant increase in activity level with *P*<.001 and *P*=.006, respectively.<br><br>Between group significant difference achieved with *P*=.001	Health behaviour score (mean ± SD)<br>Baseline (I): 1.50 ± 0.06 (C): 1.67 ± 0.09<br>24-weeks (I): 2.12 ± 0.06 (C): 1.76 ± 0.10<br>Significant difference within intervention group<br>*P*=.004<br>Significant difference between group *P*<.001	1. The service was launched for 1000 people and 911 people continued to use the app until week 24.
**Wijaya and Widiantoro[50] (2018), Taiwan, Indonesian**	n = 75 (70)<br>Indonesian international students aged ≥20 years who owns a smartphone with internet access, not participating in other training program and is literate in English.	NA	Steps/day via activity tracker (mean ± SD)<br>Baseline<br>(I): 2689.80 ± 4547.49<br>(C): 2663.98 ± 4458.58<br>10-weeks<br>(I): 7757.99 ± 8718.71<br>(C): 3704.97 ± 5217.14<br>Significant difference between group<br>*P*<.001	Self-efficacy score<br>(mean ± SD)<br>Baseline<br>(I): 75.25 ± 94.45<br>(C): 65.67 ± 87.33<br>10-weeks<br>(I): 79.95 ± 100.82<br>(C): 65.62 ± 87.43<br>Nil significant difference between group *P*=.07	

[a]T2DM: type 2 diabetes mellitus.

[b]NA: not applicable.

[c]I: intervention.

[d]C: control.

[e]HbA_{1c}: glycated haemoglobin.

[f]SDSCA: summary of diabetes self-care activities.

[g]IPAQ: international physical activity questionnaire.
